# A stochastic transcriptional switch model for single cell imaging data

**DOI:** 10.1093/biostatistics/kxv010

**Published:** 2015-03-26

**Authors:** Kirsty L. Hey, Hiroshi Momiji, Karen Featherstone, Julian R.E. Davis, Michael R.H. White, David A. Rand, Bärbel Finkenstädt

**Affiliations:** Department of Statistics, University of Warwick, Coventry CV4 7AL, UK; Warwick Systems Biology, University of Warwick, Coventry CV4 7AL, UK; Centre for Endocrinology and Diabetes, University of Manchester, Manchester M13 9PT, UK; Systems Biology Centre, University of Manchester, Manchester M13 9PL, UK; Warwick Systems Biology, University of Warwick, Coventry CV4 7AL, UK; Department of Statistics, University of Warwick, Coventry CV4 7AL, UK

**Keywords:** Bayesian hierarchical model, Birth and death processes, Gene expression, Linear noise approximation, Particle Gibbs, Reversible jump MCMC, State-space models, Stochastic reaction networks

## Abstract

Gene expression is made up of inherently stochastic processes within single cells and can be modeled through stochastic reaction networks (SRNs). In particular, SRNs capture the features of intrinsic variability arising from intracellular biochemical processes. We extend current models for gene expression to allow the transcriptional process within an SRN to follow a random step or *switch* function which may be estimated using reversible jump Markov chain Monte Carlo (MCMC). This stochastic switch model provides a generic framework to capture many different dynamic features observed in single cell gene expression. Inference for such SRNs is challenging due to the intractability of the transition densities. We derive a model-specific birth–death approximation and study its use for inference in comparison with the linear noise approximation where both approximations are considered within the unifying framework of state-space models. The methodology is applied to synthetic as well as experimental single cell imaging data measuring expression of the human prolactin gene in pituitary cells.

## 1. Introduction

In single cells, gene expression is made up of fundamentally stochastic processes (Raj and Van Oudenaarden, 2008) due to *intrinsic* and *extrinsic* variation. Here, intrinsic variability refers to the variation observed between different realizations of identical biological systems within identical environments due to the probabilistic nature of the occurrence of molecular reactions. Extrinsic variability is the intercellular variability of gene expression caused by randomness in molecular machinery within individual cells (Elowitz *and others*, 2002). Light microscopy technology used to image reporter genes has proved successful for studying stochastic temporal expression dynamics in individual live cells (Spiller *and others*, 2010). The reporter gene is inserted into cell DNA and engineered to be under the control of a native gene promoter. An important statistical problem arising from the use of reporter constructs, such as fluorescent and luminescent proteins, is to infer the unobserved transcriptional activity of the reporter, which can be related to the activity of the native gene (Finkenstädt *and others*, 2008). This activity is highly variable, occurring in stochastic pulses for many genes, including prolactin (Harper *and others*, 2011; Suter *and others*, 2011). Here we introduce a general *stochastic switch model* (SSM), to study pulsatile gene expression dynamics within single cells.

Switch models have previously been considered for inferring transcription factor interactions (Sanguinetti *and others*, 2009; Opper and Sanguinetti, 2010) and reconstructing transcription dynamics (Finkenstädt *and others*, 2008; Harper *and others*, 2011). In general, binary states are assumed (Peccoud and Ycart, 1995; Larson *and others*, 2009; Suter *and others*, 2011; Sanchez *and others*, 2013), where transcription can take only two values corresponding to the gene being active or inactive. Although the binary switch has a simple biological interpretation, the restriction to two states may not capture the full range of cellular activity as other events may influence gene regulation. The multi-state model of Jenkins *and others* (2013) was able to describe a wide range of observed dynamic patterns in gene expression including oscillatory behavior with asymmetric cycles of varying amplitude. It is the aim of this study to embed the multi-state switch model within a stochastic reaction network (SRN) for single cells whilst also introducing a measurement process to fit single cell imaging time series. Inference is challenging due to the intractability of the likelihood and we consider two approximations, the linear noise approximation (LNA) (van Kampen, 1961) and an alternative approximation that is derived specifically for the SSM. We introduce the biological motivation and model in Section 2. A brief overview of SRNs and their associated approximations is given in Section 3 with inferential techniques discussed in Section 4. Section 5 presents a simulation study while an application to data is presented in Section 6.

## 2. A stochastic switch model

**Fig. 1.**
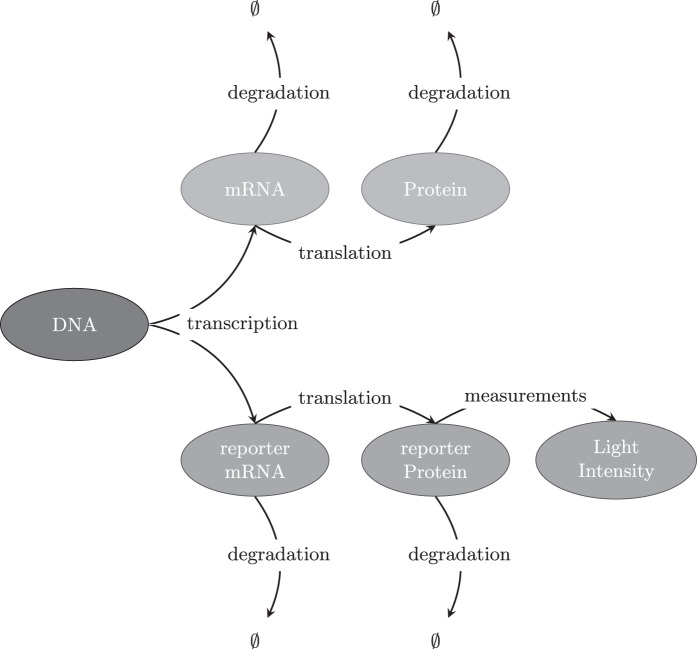
A diagrammatic representation of the transfer of information from DNA to protein through transcription and translation and its relation to the measurement process through a reporter gene construct. Specifically, the cell DNA is engineered such that the reporter gene is regulated through the same regulatory sequences as the gene of interest. Thus, transcription of the reporter gene and target gene will be highly coupled. Once transcribed, the mRNA molecules will either degrade or be translated into proteins. Note that there is no longer any coupling between the native and reporter species after transcription and thus the remaining reactions occur at differing rates. The abundance of reporter protein can be measured indirectly through microscopy techniques. Consequently, the aim of this methodology is to backcalculate from reporter protein levels, to reporter mRNA levels and infer the reporter transcriptional dynamics. This will then give a fair representation of the native transcriptional dynamics.

**Fig. 2.**
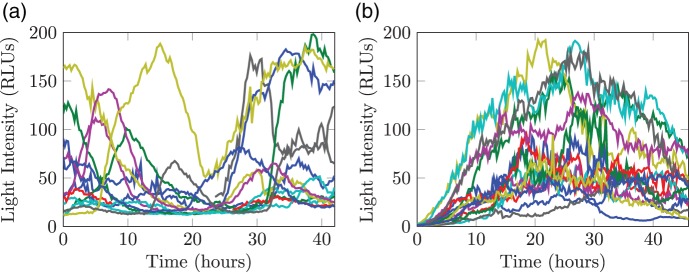
Fluorescent time course data of 15 randomly selected cells from (a) an immature (post-natal day 1.5) rat pituitary tissue slice and (b) a mature (adult male) rat pituitary slice. Measurements were taken every 15 min over 42 h.

**Fig. 3.**
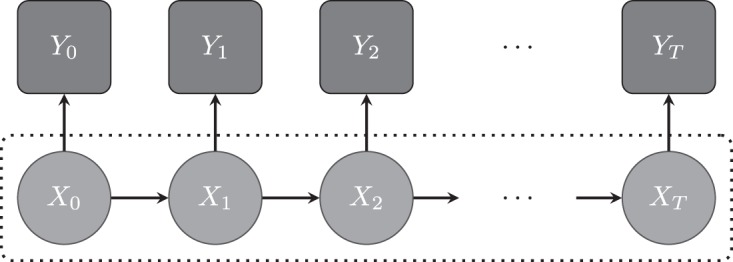
A graphical representation of the state-space model for single cell imaging data. The arrows depict conditional dependencies between nodes, where square nodes are observed variables and circular nodes are unobserved. The observed light intensities, $Y_t$, are conditionally independent given the unobserved latent states ${\textbf{X}}_t:= (M_t, P_t)$ consisting of reporter mRNA, $M_t$, and reporter protein, $P_t$, levels at time $t$. Moreover, the latent states follow a Markov jump process with ${\textbf{X}}_t$ depending only upon the previous states ${\textbf{X}}_{t-1}$.

The basic model of gene expression (Paulsson, 2005) describing the transfer of information encoded within DNA to the creation of protein molecules is given by

(2.1) \[\mathrm {DNA} \xrightarrow [\quad \quad ]{\beta (t) } \mathrm {mRNA},\quad \quad \mathrm {mRNA} \xrightarrow [\quad \quad ]{\delta _m} \emptyset ,\]

(2.2) \[\mathrm {mRNA} \xrightarrow [\quad \quad ]{\alpha } \mathrm {mRNA} + \mathrm {Protein}, \quad \quad \mathrm {Protein} \xrightarrow [\quad \quad ]{\delta _p } \emptyset ,\]

where the superscript for each reaction denotes the corresponding reaction rate. Following Jenkins *and others* (2013), we model transcription by a piecewise constant function, $\beta (t)= \beta _i$ for $t \in [s_{i-1}, s_{i})$, where changes in rates are associated with unobserved transcriptional events occurring at unknown switch times $s_1,\ldots , s_{k}$. The rates of translation, $\alpha$, and degradation, $\delta _m$ and $\delta _p$, are assumed constant. Figure 1 gives a diagrammatic representation of how the measurement process, via reporter genes, relates to native gene expression. Our aim is to backcalculate from light intensity measurements, to reporter protein levels, back to reporter mRNA levels and finally to the transcriptional dynamics of the reporter, which will relate to the transcriptional dynamics of the native gene since the reporter is under the control of the native gene promoter. Figure 2 shows fluorescent time course data for 15 randomly selected cells from samples of immature and adult rat pituitary tissue. The target gene for these data is the prolactin gene whose regulation is of physiological interest due to its important roles in mammalian reproduction and also its frequent over-production by pituitary adenomas (Featherstone *and others*, 2012). For further details of the reporter construct used and associated experimental framework; see Semprini *and others* (2009), Harper *and others* (2010) and Featherstone *and others* (2011). We assume that the observed fluorescent time course, $Y$, are indirect measurements of reporter protein levels, $Y = \kappa P + \epsilon$, $\epsilon \sim \hbox {{\textit {N}}}(0, \sigma _{\epsilon }^2)$, and are conditionally independent given the latent states, ${\textbf{X}}:= (M, P)^{\rm T}$, consisting of the unobserved reporter species, mRNA ($M$) and protein ($P$). Consequently, the system follows a state-space model (Figure 3) where ${\textbf{X}}$ is a Markov jump process given in the following section.

## 3. Stochastic reaction networks and their approximations

SRNs can be used to model systems of reactions such as (2.1) and (2.2) by Markov jump processes (MJPs). Consider a system of $\nu$ stochastic reactions involving $D$ molecular species, ${\textbf{X}}=(X_1,\ldots , X_D)^{\rm T}$, in a well-mixed environment of volume $\Omega$. The stochastic process can be represented by the set of reactions, ${\mathcal P} {\textbf{X}} \xrightarrow {{\textbf{h}}} {\mathcal Q} {\textbf{X}}$, for matrices ${\mathcal P}$ and ${\mathcal Q}$. The vector of hazards ${\textbf{h}}$, describes the rate at which each reaction occurs and in general will depend on ${\textbf{x}}$, the current state of the random vector ${\textbf{X}}$, and kinetic rates $\theta$. The vectors ${\textbf{v}}_j$ of the stoichiometric matrix $S:={\mathcal Q} - {\mathcal P}:=[{\textbf{v}}_1,\ldots , {\textbf{v}}_{\nu }]$ describe the change in state for each reaction $j$. By the law of mass action, the hazards are given by

(3.1) \[h_j({\textbf{x}}, \theta _j) = \theta _j \prod _{k=1}^D \left ( \begin {matrix}x_k\\ {\mathcal P}_{jk} \end {matrix}\right ) \quad {\rm for} \ j = 1,\ldots , \nu ,\]

where ${\mathcal P}_{jk}$ is the $jk$th element of ${\mathcal P}$ and $x_k$ is the $k$th element of the state vector ${\textbf{x}}$. Given the system is currently in state ${\textbf{x}}$, the probability of reaction $j$ occurring so that the state vector becomes ${\textbf{x}} + {\textbf{v}}_j$, in the next infinitesimal $dt$ time is given by $h_j({\textbf{x}}, \theta _j)dt$. From this, it is straightforward to derive that the next reaction to occur will be at time $t + \tau$ and of type $j$ with probability, $\mathbb {P}({\textbf{X}}(t + \tau ) = {\textbf{x}} + {\textbf{v}}_j\,|\,{\textbf{X}}(t)={\textbf{x}})={\rm e}^{-h_0({\textbf{x}}, \theta )\tau }h_j({\textbf{x}}, \theta _j),$ where $h_0({\textbf{x}}, \theta ) = \sum _{j=1}^{\nu }h_j({\textbf{x}}, \theta _j)$. This identity forms the basis of a stochastic simulation algorithm (Gillespie, 1977), from which we can generate exact sample paths of a given system. If complete data on all species and reactions were available, inference would be straightforward since the likelihood is then given by

(3.2) \[f({\textbf{x}}\, |\, \theta ) = \prod _{i=1}^n h_{j_i}({\textbf{x}}(t_i), \theta _{j_i}) \exp \left ( -\sum _{i=0}^n h_{0}({\textbf{x}}(t_i), \theta )[t_{i+1}-t_i]\right ) ,\]

where $n$ is the number of reactions that take place, $j_1,\ldots ,j_n$ is the sequence of reaction types, and $t_1,\ldots ,t_n$ are the associated timings of each reaction. However, in molecular biology, complete data paths are not available and commonly only a subset of species are measured indirectly with error.

One approach for exact inference on partially observed SRNs is to integrate out the latent reaction paths and recent attention has been focused on evaluating these high-dimensional integrals in a computationally efficient way. Andrieu *and others* (2010) show how particle Markov chain Monte Carlo (MCMC) methods can be used to perform inference on MJPs, in particular the stochastic kinetic Lotka–Volterra model, although this was found to perform poorly in low measurement error scenarios (Golightly and Wilkinson, 2011). Other approaches for inference on the exact system include a simulation-based method (Amrein and Künsch, 2012), a reversible jump (RJ) MCMC method (Boys *and others*, 2008), an implementation of uniformization (Choi and Rempala, 2012) and the MCEM$^2$ of Daigle *and others* (2012) which makes use of rare simulation techniques. Two recent examples that also consider real data are the delayed acceptance MCMC method of Golightly *and others* (2014) applied to epidemic data and the dynamic prior propagation method of Zechner *and others* (2014) who model an artificially controlled gene expression system in yeast. All these exact inference techniques assume a known scaling factor, $\kappa$, of 1 and often also known measurement error. Moreover, the techniques used are often computationally burdensome with respect to the size of data we consider (Figure 2). In a molecular biology framework, experimental methods will invariably result in a measurement process with both unknown error and scaling as the direct number of molecules is unobservable. One approach is to rescale the data based on additional experiments (Zechner *and others*, 2014). The incorporation of both unknown measurement error and scaling is non-trivial and we will consider this in some detail.

In our study we consider the feasibility of approximating the underlying MJP by approximating the transition densities, $\mathbb {P}({\textbf{x}}, t):= \mathbb {P}({\textbf{X}}(t) = {\textbf{x}}\, |\, {\textbf{X}}(0) = {\textbf{x}}_0)$, which solve the, rarely tractable, chemical master equation

(3.3) \[\frac {{\rm d}}{{\rm d} t}\mathbb {P}({\textbf{x}},t) = \sum _{j=1}^{\nu }h_j({\textbf{x}}-{\textbf{v}}_j, \theta _j) \mathbb {P}({\textbf{x}}-{\textbf{v}}_j, t) -h_j({\textbf{x}}, \theta _j)\mathbb {P}({\textbf{x}},t), \quad \mathbb {P}({\textbf{x}},0) = {\rm \mathbb {I}}[{\textbf{x}}={\textbf{x}}_0].\]

The reader is referred to Appendix A (see supplementary material available at *Biostatistics* online) for detailed derivations of each approximation.

We first note that, in the macroscopic limit, a deterministic approximation, ${\textbf{X}}^{{\rm D}}$, is given by

(3.4) \[\frac {{\rm d} {\textbf{X}}^{{\rm D}}}{{\rm d} t} = A({\textbf{X}}^{{\rm D}}):= \sum _{j=1}^{\nu } {\textbf{v}}_j h_j({\textbf{X}}^{{\rm D}}, \theta _j) = S {\textbf{h}}({\textbf{X}}^{{\rm D}}, \theta ), \quad {\textbf{X}}^{{\rm D}}(0) = {\textbf{x}}_0,\]

which may be appropriate for modeling high-level aggregate data with negligible intrinsic variability as in Jenkins *and others* (2013).

At the mesoscopic level there are two approximations that have been used to model SRNs, namely the chemical Langevin equation (CLE) and the LNA, both of which give rise to systems of SDEs. The LNA is a linearization of the master equation and always results in analytical transition densities. Derivations of varying degrees of rigour can be found (van Kampen, 1961; Wallace *and others*, 2012) with Kurtz (1971) deriving it as a central limit theorem for the underlying MJP. The LNA is specified by

(3.5) \[{\textbf{X}}^{{\rm L}}(t)= \phi (t) + \Omega ^{-1/2}\xi (t),\]

(3.6) \[\frac {{\rm d}\phi }{{\rm d} t}= A(\phi (t)), \quad {\rm d}\xi = J(\phi (t)) \xi (t) \,{\rm d} t + {B(\phi (t))}\,{\rm d} W_t,\]

where $\phi$ is the macroscopic ODE solution, ${\rm d} W_t$ are independent Wiener processes, $A$ is defined as in (3.4), $B:= \sqrt {S {\rm {\rm diag}} ({\textbf{h}}(\phi (t), \theta )) S^{\rm T}},$ and $J = (J_{ij}) = ({\partial A_j}/{\partial \phi _j})$ is the Jacobian. Since the SDE in equation (3.6) is linear with Itô representation, the transition ${\rm \mathbb {P}}(\xi (t + \tau )\,|\,\xi (t))$ is Gaussian with mean and variance (Komorowski *and others*, 2009) defined by

(3.7) \[\frac {{\rm d} \mu }{{\rm d} t} = J(\phi (t)) \mu (t), \quad \frac {{\rm d} \Sigma }{{\rm d} t} = \Sigma (t) J(\phi (t))^{\rm T} +J(\phi (t)) \Sigma (t)^{\rm T} +B(\phi (t))B(\phi (t))^{\rm T}.\]

Thus, the transition probabilities of the state vector are given by ${\rm \mathbb {P}}({\textbf{X}}^{{\rm L}}(t + \tau ) \,|\, {\textbf{X}}^{{\rm L}}(t)) = \hbox {{\textit {N}}}(\phi (t) + \Omega ^{-1/2}\mu (t + \tau ), \Omega ^{-1} \Sigma (t + \tau ))$. In the case of a linear system where $J(\phi (t)) \equiv J$ is independent of time, as in our gene transcription model, (3.7) can be simplified to give $\mu (t + \tau ) = {\rm e}^{J \tau } \xi (t)$ and $\Sigma (t + \tau ) = \int _t^{t + \tau } [{\rm e}^{J(t + \tau - s)} B(s)][{\rm e}^{J(t + \tau - s)} B(s)]^{\rm T} \,{\rm d} s$.

Both the CLE and LNA are derived in the limit as the system size $\Omega \to \infty$ with precise statements given in Kurtz (1971, 1978). Despite the LNA commonly being derived as an approximation to the CLE, Anderson and Kurtz (2011) show that in fact less stringent assumptions are required for the derivation. Inference on different transcription networks including autoregulatory and dimerization systems using the LNA are given in Ruttor and Opper (2009), Komorowski *and others* (2009), Stathopoulos and Girolami (2013), Finkenstädt *and others* (2013) and Fearnhead *and others* (2014). Although the LNA is derived in the large system size limit, these studies found reasonable performance when the system is of mesoscopic size.

Finally, we construct a further approximation for the gene expression reaction network (2.1)–(2.2), consisting of conditionally independent birth–death networks (Appendix A.4 of supplementary material available at *Biostatistics* online) given by

(3.8) \[{2} \emptyset \xrightarrow [\quad \quad \quad ]{\beta (t)} M, \quad \quad M \xrightarrow [\quad \quad \quad ]{\delta _m} \emptyset ,\]

(3.9) \[\emptyset \xrightarrow [\quad \quad \quad ]{\alpha M^* (t)} P,\quad \quad P \xrightarrow [\quad \quad \quad ]{\delta _p} \emptyset .\]

This approximation corresponds to the following factorization of the joint transition density:

(3.10) \[\begin {array}{rl} \mathbb {P}(M(t),P(t)\,|\,M(0), P(0)) & = \mathbb {P}(M(t)\,|\,M(0), P(0))\mathbb {P}(P(t)\,|\, M(t), P(0)) \\ & \approx \mathbb {P}(M(t)\,|\,M(0))\mathbb {P}(P(t)\,|\,M^* (t), P(0)). \end {array}\]

**Fig. 4.**
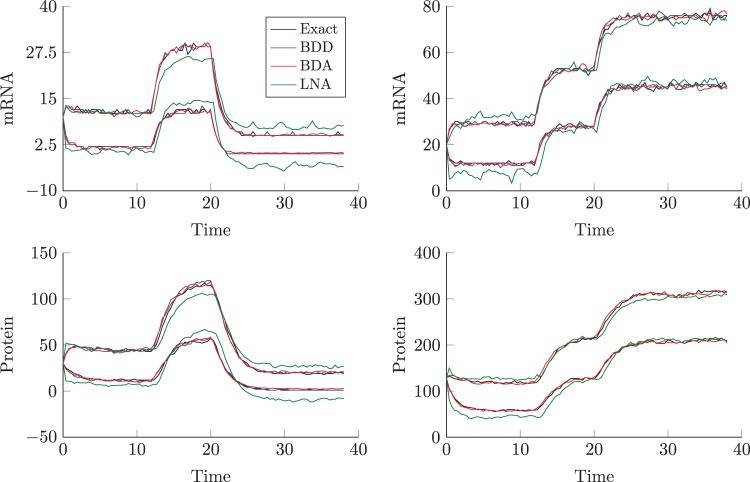
95% pointwise confidence envelopes for simulated mRNA (top) and protein (bottom) processes under the true process (black), the BDD (blue), the truncated normal BDA (red), and the LNA (green) for two different scenarios corresponding to different molecular abundances. Scenario 1 (left) is simulated from the parameters $\delta _m=1, \delta _p=0.7$, $\alpha =3, \beta _0=6, \beta _1=20, \beta _2=2$ with switches occurring at $t=12, 20$. Scenario 2 (right) is simulated with parameters $\delta _m=1, \delta _p=0.7, \alpha =3,\beta _0=20, \beta _1=40, \beta _2=60$ with switches occurring at $t=12, 20$.

Note that the exact system is obtained by setting $M^* $ to be the continuous time mRNA process, $m(t)$, while our approximation arises from setting $M^* (t):= m_t$ to be the discrete time mRNA process. Although alternative definitions of $M^* $ have been considered (Appendix A.4 of supplementary material available at *Biostatistics* online), we find $M^* (t)=m_t$ provides the best proxy to the exact continuous time process. Under this birth–death decomposition (BDD), one can obtain the exact transition densities for the two separable birth–death subsystems in (3.8)–(3.9) and note that the approximation only affects inference regarding the protein process since marginal inference for the mRNA process will be exact. The resulting transition densities are Poisson-binomial convolutions which may be approximated by a normal density truncated to the positive real line. We term this approach the birth–death approximation (BDA). The improved precision of the BDA becomes apparent in Figure 4, which shows 95% pointwise simulation envelopes for different scenarios. In all scenarios, the BDD and BDA envelopes for both mRNA and protein are closer to the true envelopes with the truncated normal approximation modeling the skewness at low molecular numbers better than the symmetric LNA. The LNA improves as molecular numbers increase although consistently overestimates the variance of the true process for low numbers and will consequently be likely to miss switch points in the transcriptional profiles. This empirical validation, supported by Appendix A.4 (see supplementary material available at *Biostatistics* online), reinforces the intuition that the BDA may be a preferable approximation for systems of low molecular levels.

## 4. Inference

In the presence of a measurement process, state-space models (as depicted in Figure 3) provide a framework for modeling SRNs and their approximations. Specifically, we have

(4.1) \[X_{t+1} \sim h(x_{t+1}\,|\,x_t, \theta ), \quad Y_t \sim g(y_t\,|\,x_t, \theta ) ,\]

where $h$ is the transition density of the approximating SRN and $g$ is the density of the measurement process. For ease of notation, we have dropped any explicit dependence on time, i.e. the sequence of observations $(Y_0,\ldots , Y_T)$ are assumed to occur at arbitrary times, $(0, t_1,\ldots , t_T)$ and are equivalent to $(Y(0), Y(t_1),\ldots , Y(t_T))$ in the notation of Section 3. Moreover, we let ${\textbf{y}}$ denote the vector of observed data points $y_0,\ldots , y_T$ and let $\theta$ denote the unknown parameter vector.

We now investigate the performance of the LNA and BDA for approximating the posterior $f(\theta \,|\,{\textbf{y}})$ of the underlying SRN. The data likelihood is given by the marginal density,

(4.2) \[f({\textbf{y}}\,|\,\theta ) = \int _{{\textbf{x}}} f({\textbf{y}}, {\textbf{x}}\,|\,\theta )\, {\rm d}{\textbf{x}} = \int _{{\textbf{x}}} h(x_0\,|\,\theta )g(y_0\,|\,x_0, \theta ) \prod _{t=1}^{\rm T} h(x_t\,|\,x_{t-1}, \theta ) g(y_t\,|\,x_t, \theta ) \,{\rm d}{\textbf{x}}.\]

Under the LNA with Gaussian measurement error, the above integral can be computed explicitly using the Kalman methodology (see Appendix B.1 of supplementary material available at *Biostatistics* online). Under the BDA, equation (4.2) is intractable and one instead targets the joint posterior $f(\theta ,{\textbf{x}}\,|\,{\textbf{y}})$ through the following Gibbs sampler (GS):

1. Sample the parameter vector $\theta$ from $f(\theta \,|\,{\textbf{y}},{\textbf{x}})$.
2. Sample the latent states, ${\textbf{x}}$, from the conditional density, $f({\textbf{x}}\,|\,{\textbf{y}}, \theta )$.

*Parameter inference*. In order to sample $\theta$ from either $f(\theta \,|\, {\textbf{y}}, {\textbf{x}})$ or $f(\theta \,|\, {\textbf{y}})$, depending on the approximation used, we construct an appropriate MCMC sampler. In particular, inference about $\theta$ includes inference on the number, $k$, and position, $s_1,\ldots , s_k$ of switches as well as the associated kinetic parameters $(\beta _0,\ldots , \beta _k, \alpha , \delta _m, \delta _p)$, the measurement parameters, $(\kappa , \sigma _{\epsilon }^2)$ and the initial state of the latent molecular processes, $(M_0, P_0)$. Details of all prior distributions are given in Appendix C of supplementary material available at *Biostatistics* online. Since the dimension of $\theta$ varies with the number of switches, we employ a RJ scheme (Green, 1995) to sample across the differing dimensions. In particular, at each iteration of the MCMC, we propose one of three possible moves: (1) add a switch, (2) delete a switch, and (3) move a switch. Further details can be found in Appendix D of supplementary material available at *Biostatistics* online.

Owing to the high dimensionality of the integral in (4.2), there is a strong correlation between different model parameters. In order to sample efficiently, we reparameterize $\tilde {\alpha }:=\kappa \alpha$ and $\tilde {P}_t:= \kappa P_t$ and target the posterior of the log-parameters ($\log \theta$). Efficiency was further increased through the adaptive scheme of Haario *and others* (2001). Specifically, these log-parameters were sampled in two blocks where proposals are drawn from a multivariate Gaussian centered at the previous value, with the covariance matrix proportional to the covariance of the Markov chains. This adaptation results in an ergodic Markov chain provided the target density is bounded from above and has bounded support.

*Inferring the latent states*. There are many ways one can perform the filtering procedure in step 2 of the GS (see Fearnhead, 2011 for a review). Under the BDA, we found a conditional sequential Monte Carlo particle filter (Andrieu *and others*, 2010) to perform well. The approach is based on forward simulations to sequentially approximate the filtering density, $f(x_t\,|\,y_{0:t}, \theta )$ and can be applied to very general state-space models that are not necessarily linear or Gaussian. The filtering density is approximated by $f^{N_p}(x_t\,|\,y_{0:t}, \theta ) = \sum _{i=1}^{N_p} w^{(i)}_t \delta _{x^{(i)}_t}$, where $\delta _x$ is a delta function centered at $x$, and $w^{(i)}_t$ are importance weights. Given the approximate filtering density $f^{N_p}({\textbf{x}}\,|\,{\textbf{y}}, \theta ):=f^{N_p}(x_{0:T}\,|\,y_{0:T}, \theta )$, one can obtain a sample of the latent states ${\textbf{x}}$ as required and the resulting algorithm is termed *Particle Gibbs* (Andrieu *and others*, 2010). Further details of the algorithm and proposal densities used for the BDA can be found in Appendix B.2 (see supplementary material available at *Biostatistics* online).

*Hierarchical modeling*. In order to incorporate as much information as possible into the algorithm, informative prior distributions are desirable. In the example of single cell imaging data, additional experiments can be performed to obtain estimates of the degradation parameters, $\delta _m$ and $\delta _p$. A hierarchical structure can be used to aid in the identification of the remaining parameters since a dataset will typically consist of multiple time series from the same experiment (Finkenstädt *and others*, 2013). Let ${\textbf{y}}^{(i)}$ denote the observed time series for cell $i$, and $\theta ^{(i)}:=(\beta ^{(i)}(t), \alpha ^{(i)}, \delta ^{(i)}_m, \delta ^{(i)}_p, \kappa ^{(i)}, \sigma _{\epsilon }^{(i)}, M_0^{(i)}, P_0^{(i)})$, the vector of parameters, for $i=1,\ldots ,N$. We assume a log-normal hierarchical structure for translation rates, $\log \alpha ^{(i)} \sim \hbox {{\textit {N}}}(\mu _{\alpha },\sigma ^2_{\alpha })$, and measurement parameters, $\log \kappa ^{(i)} \sim \hbox {{\textit {N}}}(\mu _{\kappa },\sigma ^2_{\kappa })$, $\log \sigma _{\epsilon }^{(i)} \sim {\textit {N}}(\mu _{\sigma },\sigma ^2_{\sigma }),$ which allows a conjugate update of the hyper-parameters (Appendix E of supplementary material available at *Biostatistics* online).

Specifying a hierarchical model for the transcription rates $\beta :=(\beta ^{(1)},\ldots , \beta ^{(N)})$, where $\beta ^{(i)}:=(\beta _0^{(i)},\ldots , \beta _k^{(i)})$ is the vector for each cell $i$, is less straightforward. To use the same specification as above would dilute the effect of switching events since all rates would be shrunk to a single distribution. On the other hand, vague proper priors are not a feasible option since it gives too much prior probability to the zero switch model (Green, 1995). As an alternative, we specify a hierarchical mixture model with $\log \beta ^{(i)} \sim \sum _{m=1}^M w_{\beta _m} \hbox {{\textit {N}}}(\mu _{\beta _m}, \sigma _{\beta _m}^2)$, which reduces the hierarchical shrinkage. Without resorting to a second RJ, it is necessary to specify the number of components in advance. One could choose several candidates and perform model selection *a posteriori*, although we found two components sufficient to capture the variability in the data, which is supported by the biological hypothesis that transcription will typically occur at either a high or low rate. Simulations showed that if the rates truly come from a single component, then this is elicited from a two-component specification with one weight estimated to be very low.

The hyper-parameters $\vartheta := (\mu _{\alpha }, \sigma _{\alpha }^2, \mu _{\kappa }, \sigma _{\kappa }^2, \mu _{\sigma }, \sigma _{\sigma }^2, \mu _{\beta }, \sigma _{\beta }^2, w_{\beta })$ are assigned uninformative prior densities (Appendix C of supplementary material available at *Biostatistics* online) and are estimated in addition to each $\theta ^{(i)}$.

Consequently, the algorithm specification for sampling from the full posterior $f(\theta ^{(1)},\ldots , \theta ^{(N)}, \vartheta \,|\, {\textbf{y}}^{(1)},\ldots , {\textbf{y}}^{(N)})$ has the following structure where additional steps required only under the BDA are given in italics:

1. Initialization
  - Initialize parameters, $\theta$.
  - *Initialize the latent states $M^{(i)}_1,\ldots ,M^{(i)}_T, \tilde {P}^{(i)}_1,\ldots , \tilde {P}^{(i)}_T$*.
2. Update hyper-parameters, $\vartheta$, from the full conditional, $f(\vartheta \,|\, \theta ^{(1)},\ldots , \theta ^{(N)}, {\textbf{y}}^{(1)},\ldots , {\textbf{y}}^{(N)}) = f(\vartheta \,|\, \theta ^{(1)},\ldots , \theta ^{(N)})$.
3. For cell $i=1,\ldots ,N$, sample $\theta ^{(i)}$ *and the latent states,*
  - update the log transcriptional step function by RJ step;
  - sample $\log (\beta ^{(i)}_0,.., \beta ^{(i)}_k, \delta ^{(i)}_m, \delta ^{(i)}_p, M^{(i)}_0)$ parameters by a random walk Metropolis–Hastings (MH) step;
  - sample $\log (\tilde {\alpha }^{(i)}, \kappa ^{(i)}, \sigma _{\epsilon }^{(i)}, \tilde {P}^{(i)}_0)$ parameters by a random walk MH step;
  - *update the latent states, $M^{(i)}_1,\ldots ,M^{(i)}_T, \tilde {P}^{(i)}_1,\ldots , \tilde {P}^{(i)}_T$, by a particle Gibbs step.*
4. Repeat steps 2 and 3 until convergence.

## 5. Simulation study

In order to investigate the performance of the LNA and BDA, we perform a comprehensive simulation study where data were generated from the exact MJP via a stochastic simulation algorithm (Gillespie, 1977). The synthetic data were constructed to replicate the main features observed in the data (Figure 2) with further details given in Appendix F (see supplementary material available at *Biostatistics* online). Applying both the LNA and BDA models to these data, it was found that informative priors for the degradation parameters were essential in order to identify both the transcriptional profile, $\beta (t)$, and translation rate, $\alpha$. We therefore imposed informative prior distributions, $\log \delta _m \sim \hbox {{\textit {N}}}(\mu _{\delta _m}, \sigma ^2_{\delta _m})$ and $\log \delta _p \sim \hbox {{\textit {N}}}(\mu _{\delta _p}, \sigma ^2_{\delta _p}),$ where the hyper-parameters were fixed at the true values. Analyses showed that, under the BDA, the scaling parameter, $\kappa$, remained unidentifiable in the majority of simulations. We hypothesize this is because, under the BDA, we are targeting an extended space by explicitly sampling the latent states. To our knowledge, there has been no application within this extended framework that has been able to incorporate a scaling parameter in the measurement equation. We hence consider two scenarios under the BDA: (1) $\kappa$ is fixed at the true value and (2) $\kappa$ is fixed at the posterior median obtained from the LNA.

The simulation study was coded in MATLAB^®^ and typically took 10–32 h to run on a standard PC under the LNA, for 200–700 K iterations. Despite the fact that the BDA methodology is computationally faster to run per MCMC iteration, due to the high autocorrelation in the chains and poorer mixing properties, we found it would take $\sim$1–3 million iterations to sufficiently explore the posterior, which could take 20–40 h. This is unsurprising since the BDA methodology requires the sampling of all latent states in addition to the parameter vector. For all scenarios under the BDA, 100 particles were used to give a sufficient number of independent samples in the particle filter. Comparing the simulation results in Appendix F (see supplementary material available at *Biostatistics* online), we find the BDA often produces tighter credible intervals. In addition, in some scenarios, the BDA is better able to identify $\alpha$ and $\beta$, which are highly correlated, whereas the LNA identifies the product $\alpha \beta$. The hierarchical structure greatly aids this identifiability and, moreover, also enables the algorithm to differentiate between intrinsic variability and transcriptional switches.

Prior estimation of the degradation parameters is essential and, moreover, the precision of these priors influences the posterior inference. Typically, 10–15 time series consisting of around 100 observations are sufficient to inform the hierarchy. More cells may be included in the hierarchy at an increased computational cost, with our methods having been successfully applied to datasets of 100 or more cells consisting of $\sim$190 time points each.

## 6. Application to data

**Fig. 5.**
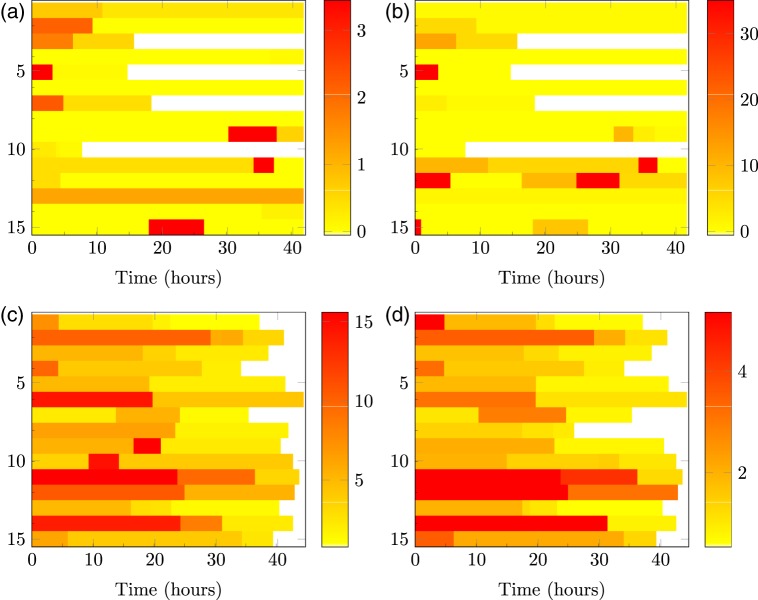
Heatmaps of the posterior transcriptional profiles for (a) the immature tissue sample and (b) the mature tissue sample calculated under the LNA. (c) and (d) are calculated under the BDA. Each row within a heatmap corresponds to a separate cell and the color is indicative of the posterior transcription rate (calculated as mRNA molecules per hour). To obtain the posterior profiles, we extract the marginal distribution of the number and position of switch times. Conditional on these times, the posterior rates are then extracted from the MCMC output.

**Fig. 6.**
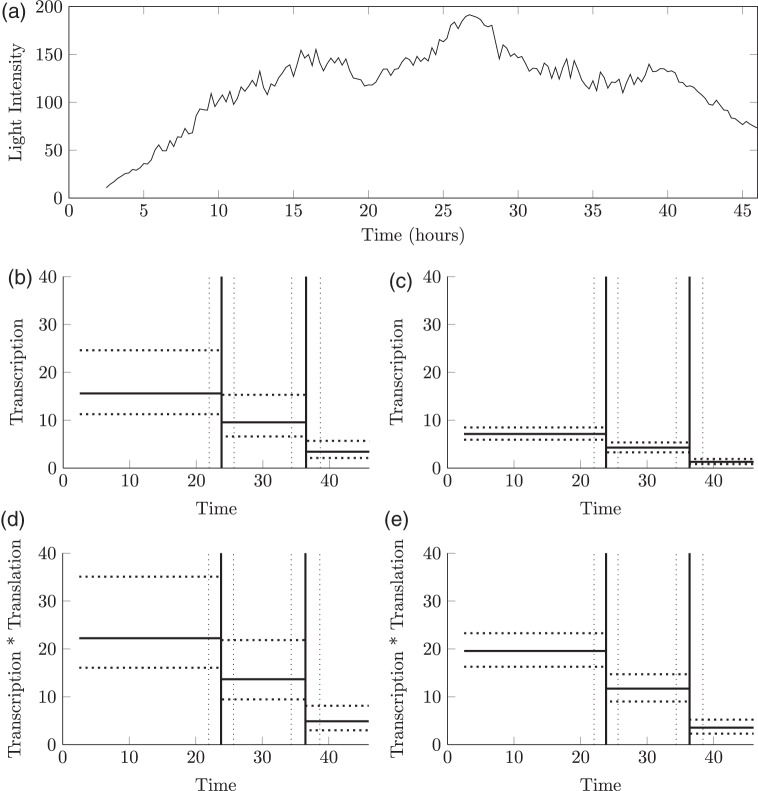
(a) The raw time course data for a single cell from the mature tissue sample, with the backcalculated transcriptional profile given in (b) under the LNA and in (c) under the BDA. The reparameterized profile of transcription $\times$ translation, $\alpha \beta$ is given in (d) for the LNA and (e) for the BDA. Dashed lines represent the 95% credible intervals about the posterior median transcriptional switches (vertical lines) and transcriptional rates (horizontal lines).

To apply our methods to the data shown in Figure 2, priors over the reporter degradation rates are obtained from Finkenstädt *and others* (2013). We first apply the LNA and then apply the BDA with $\kappa$ fixed at the posterior median obtained from the LNA. For real data, significantly more iterations were required to fully explore the posterior under the BDA (8 and 4.5 million iterations for the two datasets given in Figures 2(a) and (b)) compared to the LNA (300 and 900 K, respectively). The estimated transcriptional profiles for both datasets are given in Figure 5. Both tissues exhibit dynamic switching behavior with multiple switching events occurring throughout the time course that would not be exhibited under the traditional binary model. Figure 6 shows a single backcalculation under both the LNA and BDA along with 95% credible intervals of the posterior switch times and transcription rates. This example typifies the two methods, where although the estimated transcription rates differ, the product of translation and transcription, $\alpha \beta$, along with the estimated switch times are comparable with tighter intervals obtained under the BDA. The model fit was assessed through the analysis of recursive residuals of the one-step ahead predictive distribution and are shown in Appendix G (see supplementary material available at *Biostatistics* online) with no departure from the model assumptions detected, indicating that the SSM under both the LNA and BDA fits the data well.

More extensive biological analyses of these and other datasets will be presented in forthcoming work, including analyses of the inter-switch times which can provide further insight into gene regulation. For instance, if the inter-switch time distribution departs from exponential behavior it may indicate the presence of a refractory period as introduced in Harper *and others* (2011).

## 7. Discussion

In this study, we have proposed a general methodology for inferring transcriptional regulation for data obtained through single cell imaging techniques. The underlying biological model is flexible enough to describe a wide range of behaviors that cannot be captured by the traditional binary model and can be estimated reliably through a RJ scheme. In order to achieve the above, we consider two approximations to the true stochastic system. With a slight loss in precision, the LNA has the advantage both in terms of computational speed, through the use of the Kalman methodology, and also its ability to identify the scaling parameter of the measurement process. This parameter is of interest as it allows one to obtain an estimate of the underlying system size. However, since the BDA can give a more accurate representation of the stochastic system, it may suggest the use of this in conjunction with the LNA estimate of $\kappa$. The BDA, although more expensive than the LNA, is still considerably cheaper than the exact methods reviewed within this paper as we continue to work with the underlying transition densities albeit through a normal approximation. It therefore provides a realistic alternative to both the LNA and exact approaches when inferring systems of very small molecular numbers. The BDA is specific to our gene expression model, however, many different SRNs can be approximated by sequences of conditionally independent birth–death reactions and a similar approach may be more widely applied. Despite the theoretical advantages of the more exact BDA, for practical implementation on large datasets we consider the LNA to give reasonable approximations in realistic computational run time. For further increases in computational time one may consider approximate inference methods such as variational Bayes (see, for example, Opper and Sanguinetti, 2010; Opper *and others*, 2010).

This paper has focussed on the implementation of a SSM for transcription. We have shown how these methods may readily be applied to data whereupon further analysis of the posterior transcriptional profiles may give insight into the underlying mechanisms of gene expression. This is in contrast to *a priori* assuming a specific regulatory network, which, to ensure model identifiability, often requires a steady-state assumption (Tkačik and Walczak, 2011) that does not correctly model the intrinsic noise (Thomas *and others*, 2012).

The SSM provides an approach which is both flexible and scientifically interpretable. The natural hierarchical structure enables the differentiation of intrinsic variability and transcriptional switches. This has been exemplified through the application to the prolactin gene where our posterior inference shows a clear dynamic switching regime for many different transcriptional levels. Moreover, the prolactin gene provides a good example for modeling gene expression through stochastic processes with random transcriptional pulses as it exemplifies features found in many different genes (Suter *and others*, 2011).

## Supplementary material

Supplementary Material is available at http://biostatistics.oxfordjournals.org.

## Funding

K.L.H. is supported by the Engineering and Physical Sciences Research Council (ASTAA1112.KXH), H.M., D.A.R. by Wellcome Trust Grant (RSMAA.3020.SRA), and K.F., J.R.E.D., M.R.H.W. by Wellcome Trust Grant (67252). Funding to pay the Open Access publication charges for this article was provided by RCUK and COAF.

## Supplementary Material

Supplementary Data
