# Supplementary material for: A stochastic transcriptional switch model for single cell imaging data
Source: Biostatistics. 2015 Mar 26;16(4):655–69. doi: 10.1093/biostatistics/kxv010 (PMC4570576; doi:10.1093/biostatistics/kxv010)
Supplement: Supplementary Data [file supp_16_4_655__index.html]

A stochastic transcriptional switch model for single cell imaging data — Supplementary Data 

# A stochastic transcriptional switch model for single cell imaging data

## Supplementary Data

Supplementary Data

**Files in this Supplementary Material:**

- Supplementary Data - Pdf file
