# Supplementary material for: A stochastic transcriptional switch model for single cell imaging data
Source: Biostatistics. 2015 Mar 26;16(4):655–69. doi: 10.1093/biostatistics/kxv010 (PMC4570576; doi:10.1093/biostatistics/kxv010)
Supplement: Supplementary Data [file supp_kxv010_kxv010supp.pdf]

Biostatistics (2014), **0**, 0, *pp.* 1–51
doi:10.1093/biostatistics/appendices˙resubmission

Supplementary Material: Inference for a transcriptional stochastic switch model from single cell imaging data

Kirsty Hey[1*], Hiroshi Momiji[2], Karen Featherstone[3], Julian Davis[3], Mike White[4],

David Rand[2], Bärbel Finkenstädt[1*]

[1] *Department of Statistics, University of Warwick, Coventry, CV4 7AL,UK*
[2] *Warwick Systems Biology, University of Warwick, Coventry, CV4 7AL, UK*
[3] *Endocrinology and Diabetes Research Group, University of Manchester, Manchester, UK*
[4] *Systems Biology Centre, University of Manchester, Manchester, UK*

K.L.Hey@warwick.ac.uk, B.F.Finkenstadt@warwick.ac.uk

*To whom correspondence should be addressed.

© The Author 2014. Published by Oxford University Press. All rights reserved. For permissions, please e-mail: journals.permissions@oup.com

This supplementary material includes greater technical detail regarding the methods used in the main paper. Specifically, Section A presents detailed derivations of the stochastic reaction network approximations referred to in the main paper. Methods for inference for state space models, in particular the Kalman and particle filters are described in Section B with specific details regarding implementation of the methodologies used in the main paper. All prior specifications are given in Section C. The specification of the reversible jump methodology is given in Section D and Section E describes the conjugate update of the hierarchical parameters. Further details of the simulation study are given in Section F and finally, Section G gives details regarding the data application used in the main paper.

APPENDIX

A. Stochastic Reaction Network Approximations

Stochastic reaction networks can be used to model systems of reactions by Markov jump processes (MJPs), see for example Wilkinson (2011). Consider a system of ν stochastic reactions involving D molecular species, $\mathbf{X} = (X_1, ..., X_D)^T$ in a well-mixed environment of volume Ω. The stochastic process can be represented by the set of reactions,

$$\mathcal{P}\mathbf{X} \xrightarrow{\mathbf{h}} \mathcal{Q}\mathbf{X},$$

for matrices $\mathcal{P}$ and $\mathcal{Q}$. The vector $\mathbf{h}$, is the vector of hazard functions describing the rate at which each reaction occurs and $S := \mathcal{Q} - \mathcal{P} := [v_1, ..., v_\nu]$ is the stoichiometric matrix. The vectors v_j, describe the corresponding change in state for each reaction j. In general, each hazard function will depend on the state of the system, $\mathbf{x}$, and the associated kinetic rate of the reaction, denoted by θ. By the law of mass action, the hazard functions are given by,

$$h_j(\mathbf{X}, \theta_j) = \theta_j \prod_{k=1}^{D} \binom{x_k}{\mathcal{P}_{jk}}, \quad \text{for} \quad j = 1, ..., \nu, \tag{A.1}$$

where $\mathcal{P}_{jk}$ is the jkth element of $\mathcal{P}$ and x_k is the kth element of the state vector $\mathbf{x}$.

In order to define the limiting approximations, we let $\mathbf{X}^{(\Omega)} := \mathbf{X}/\Omega$ denote the concentration of $\mathbf{X}$, and in particular, $\mathbf{X}^{(\Omega)}$ satisfies the same SRN as $\mathbf{X}$ with hazard rates given by $h^{(\Omega)}$ where,

$$\begin{aligned} h_j^{(\Omega)}\left(\mathbf{X}^{(\Omega)},\theta_j\right) &= \Omega h_j\left(\mathbf{X}^{(\Omega)},\theta_j\right) \\ &= \Omega^{o_j-1} h_j(\mathbf{X},\theta_j) \\ &= \theta_j \Omega^{o_j-1} \prod_{k=1}^{D} \binom{x_k}{\mathcal{P}_{jk}}, \end{aligned}$$

where the order of each reaction, $o_j := \sum_{i=1}^{D} \mathcal{P}_{ij}\,\mathbb{I}[\mathcal{P}_{ij} \neq 1]$, is defined to be the number of reactant species (Wilkinson, 2011) .

It is useful in the following to express the Markov Jump process in terms of the following Poisson process. Namely, for fixed τ the following equality holds,

$$\begin{aligned} \mathbf{X}(t+\tau) &= \mathbf{X}(t) + \sum_{j=1}^{\nu} \mathbf{v}_j \mathcal{K}_j, \\ \mathcal{K}_j &\sim Pois\bigg(\int_t^{t+\tau} h_j(\mathbf{X}(s),\theta_j)\mathrm{d}s\bigg), \end{aligned} \tag{A.2}$$

where $\mathcal{K}_j$ is the number of type j events occurring. Equivalently, the rescaled concentration process $\mathbf{X}^{(\Omega)} := \frac{1}{\Omega}\mathbf{X}$ satisfies the Poisson process,

$$\begin{aligned} \mathbf{X}^{(\Omega)}(t+\tau) &= \mathbf{X}^{(\Omega)}(t) + \tfrac{1}{\Omega} S \mathcal{K}^{(\Omega)}, \\ \mathcal{K}^{(\Omega)} &\sim Pois\bigg(\int_t^{t+\tau} \Omega \mathbf{h}(\mathbf{X}^{(\Omega)}(s),\theta)\mathrm{d}s\bigg), \end{aligned} \tag{A.3}$$

where S is the stoichiometric matrix, $\mathcal{K}^{(\Omega)}$ is a vector of Poisson random variables and $\mathbf{h} = (h_1, ..., h_\nu)^T$ is the vector of hazard rates.

A.1 *Macroscopic limit*

The direct deterministic analogue, $\mathbf{X}^{\mathrm{D}}(t)$, of the stochastic model, $\mathbf{X}(t)$, is given by the conditional expectation of the stochastic process given its history (Chesson, 1978),

$$\mathbf{X}^{\mathrm{D}}(t+\tau) = \mathrm{E}\left[\mathbf{X}(t+\tau)|\mathbf{X}(t) = \mathbf{X}^{\mathrm{D}}(t)\right] = \mathbf{X}^{\mathrm{D}}(t) + \int_t^{t+\tau} S\mathbf{h}(\mathbf{X}^{\mathrm{D}}(s),\theta)\ \mathrm{d}s. \tag{A.4}$$

This can be rewritten in the equivalent ODE for $\mathbf{X}^{\mathrm{D}}$, often referred to as the reaction rate equation (RRE) or macroscopic limit,

$$\frac{\mathrm{d}\mathbf{X}^{\mathrm{D}}}{\mathrm{d}t} = A(\mathbf{X}^{\mathrm{D}}) := \sum_{j=1}^{\nu} \mathbf{v}_j h_j(\mathbf{X}^{\mathrm{D}}, \theta_j) = S\mathbf{h}(\mathbf{X}^{\mathrm{D}}, \theta), \tag{A.5}$$

$$\mathbf{X}^{\mathrm{D}}(0) = \mathbf{x}_0. \tag{A.6}$$

Kurtz (1970) and Anderson and Kurtz (2011) show, using the law of large numbers, that this ODE can be derived as the limit of $\mathbf{X}^{(\Omega)}$ as $\Omega \to \infty$,

$$\mathbf{X}^{(\Omega)}(t+\tau) \to \mathbf{X}^{(\Omega)}(t) + \int_t^{t+\tau} S\mathbf{h}(\mathbf{X}^{(\Omega)}(s), \theta)\mathrm{d}s =: \mathbf{X}^{\mathrm{D}}(t+\tau), \quad \text{as } \Omega \to \infty.$$

As a consequence, for sufficiently large systems, intrinsic variability can be assumed to vanish and a deterministic ODE model can be used.

For example, letting $\mathbf{X}^{\mathrm{D}} := \left(M^{\mathrm{D}}, P^{\mathrm{D}}\right)^T$, the reaction rate equations of the gene transcription model (2.1)-(2.2) are given by,

$$M^{\mathrm{D}}(t+\tau) = M^{\mathrm{D}}(t) + \int_t^{t+\tau} \beta(s) - \delta_m M^{\mathrm{D}}(s)\mathrm{d}s,$$
$$P^{\mathrm{D}}(t+\tau) = P^{\mathrm{D}}(t) + \int_t^{t+\tau} \alpha M^{\mathrm{D}}(s) - \delta_p P^{\mathrm{D}}(s)\mathrm{d}s.$$

Equivalently, formulating as an ODE,

$$\frac{\mathrm{d}}{\mathrm{d}t}\begin{pmatrix} M^{\mathrm{D}}(t) \\ P^{\mathrm{D}}(t) \end{pmatrix} = \begin{pmatrix} \beta(t) - \delta_m M^{\mathrm{D}}(t) \\ \alpha M^{\mathrm{D}}(t) - \delta_p P^{\mathrm{D}}(t) \end{pmatrix}.$$

A.2 *Chemical Langevin Equation*

The chemical Langevin equation was first derived in the chemical physics literature in Gillespie (2000). We will follow this heuristic derivation although a more rigourous treatment can be found in Anderson and Kurtz (2011).

Assuming τ is chosen to be small enough such that $h_j(\mathbf{X}^{(\Omega)}, \theta_j)$ can be considered constant over the interval $[t, t+\tau)$ $\forall j$, known as the *first leap condition* (Gillespie, 2000), the updating

equation (A.3) becomes,

$$\mathbf{X}^{(\Omega)}(t+\tau) \approx \mathbf{X}^{(\Omega)}(t) + \tfrac{1}{\Omega} S \tilde{\mathcal{K}}^{(\Omega)}, \tag{A.7}$$

$$\tilde{\mathcal{K}}^{(\Omega)} \sim Pois(\mathbf{h}^{(\Omega)}(\mathbf{X}^{(\Omega)}(t), \theta)\tau),$$

where the integrand in the Poisson rate has been replaced by a constant. Under the *second leap condition*, namely $\Omega h_j(\mathbf{X}^{(\Omega)}(t), \theta_j)\tau$ is large $\forall j$, this Poisson random variate can be replaced by a normal density yielding, the (chemical) Langevin equation,

$$\mathbf{X}^{\mathrm{C}}(t+\tau) = \mathbf{X}^{\mathrm{C}}(t) + \tau S \mathbf{h}(\mathbf{X}^{\mathrm{C}}, \theta) + \sqrt{\tau}\sqrt{S \,\mathrm{diag}\left(\mathbf{h}(\mathbf{X}^{\mathrm{C}}, \theta)\right) S^T}\, \mathcal{Z} \tag{A.8}$$

$$\mathcal{Z} \sim N(0, I_D).$$

Formulating the CLE in terms of the concentration process $\mathbf{X}^{(\Omega)}$, we notice that the second leap condition is satisfied as the system size $\Omega \to \infty$.

Taking the limit as $\tau \to 0$, equation (A.8) can be expressed by the following Itô diffusion process (Gardiner, 1985),

$$\mathrm{d}\mathbf{X}^{\mathrm{C}} = A(\mathbf{X}^{\mathrm{C}})\mathrm{d}t + \sqrt{B(\mathbf{X}^{\mathrm{C}})}\mathrm{d}\mathbf{W}_t. \tag{A.9}$$

$$A(\mathbf{X}^{\mathrm{C}}) := \sum_{j=1}^{\nu} \mathbf{v}_j h_j(\mathbf{X}^{\mathrm{C}}, \theta_j) = S\mathbf{h}(\mathbf{X}^{\mathrm{C}}, \theta), \tag{A.10}$$

$$B(\mathbf{X}^{\mathrm{C}}) := S \,\mathrm{diag}\left(\mathbf{h}(\mathbf{X}^{\mathrm{C}}, \theta)\right) S^T. \tag{A.11}$$

Returning to the linear gene transcription model of (2.1)-(2.2), the corresponding CLE, $\mathbf{X}^{\mathrm{C}} := \left(M^{\mathrm{C}}, P^{\mathrm{C}}\right)^T$, will satisfy (A.9) with,

$$A = \begin{pmatrix} \beta(t) - \delta_m M^{\mathrm{C}}(t) \\ \alpha M^{\mathrm{C}}(t) - \delta_p P^{\mathrm{C}}(t) \end{pmatrix}, \qquad B = \begin{pmatrix} \beta(t) + \delta_m M^{\mathrm{C}}(t) & 0 \\ 0 & \alpha M^{\mathrm{C}}(t) + \delta_p P^{\mathrm{C}}(t) \end{pmatrix}.$$

The CLE has been used extensively for inference within SRNs, (Golightly and Wilkinson, 2005, 2011; Heron *and others*, 2007). Despite this there are several drawbacks, not least of all, that the transition density often remains intractable. Moreover, in practice, data are measured at discrete time intervals that cannot be assumed to satisfy the first leap condition and consequently,

one is often required to integrate over the unobserved processes between observations as in Heron *and others* (2007).

A.3 *Linear Noise Approximation*

The linear noise approximation (LNA) is a linearisation of the master equation and always results in analytical transition densities. Derivations of varying degrees of rigour can be found in numerous sources (see for example, van Kampen (1961); Kurtz (1971); Wallace *and others* (2012)). Van Kampen's system size expansion evolves from the Ansatz,

$$\mathbf{X}^{\mathrm{L}}(t) = \phi(t) + \Omega^{-1/2}\xi(t), \tag{A.12}$$

where ϕ is a deterministic path, ξ a stochastic fluctuation and Ω is the size of the system. In particular, $\phi := \mathbf{X}^{\mathrm{D}}$, is the macroscopic solution. The derivation then proceeds via a second order Taylor expansion about the master equation for the solution (A.12). Kurtz (1971) on the other hand, provides a rigourous foundation for the LNA with a detailed application to SRNs given in Anderson and Kurtz (2011), which we follow here. In particular, the LNA is derived as a central limit theorem to the Poisson process given in equation (A.3). To see this, we consider $V^{(\Omega)} := \sqrt{\Omega}(\mathbf{X}^{(\Omega)} - \phi)$ to be the deviation between the Poisson process, $\mathbf{X}^{(\Omega)}$, and the macroscopic limiting process, ϕ,

$$\begin{aligned}
V^{(\Omega)}(t+\tau) &= \sqrt{\Omega}\left(\mathbf{X}^{(\Omega)}(t+\tau) - \phi(t+\tau)\right) \\
&= \sqrt{\Omega}\left(\mathbf{X}^{(\Omega)}(t) - \phi(t)\right) + ... \\
&\qquad ... + \sqrt{\Omega}\left(\tfrac{1}{\Omega} S\mathcal{K}^{(\Omega)} - S\int_t^{t+\tau} \mathbf{h}(\phi(s),\theta)\,\mathrm{d}s\right) \\
&= V^{(\Omega)}(t) + \tfrac{1}{\sqrt{\Omega}} S\mathcal{K}^{(\Omega)} - \sqrt{\Omega}\int_t^{t+\tau} A(\phi(s))\,\mathrm{d}s,
\end{aligned}$$

where A is defined as in equation (A.5). Thus we have,

$$V^{(\Omega)}(t+\tau) - V^{(\Omega)}(t) = \tfrac{1}{\sqrt{\Omega}} S\mathcal{K}^{(\Omega)} - \sqrt{\Omega}\int_t^{t+\tau} A(\phi(s))\ \mathrm{d}s, \tag{A.13}$$
$$\mathcal{K}^{(\Omega)} \sim \mathit{Pois}\bigg(\int_t^{t+\tau} \Omega\mathbf{h}(\mathbf{X}^{(\Omega)}(s),\theta)\ \mathrm{d}s\bigg).$$

Using the trick of adding zero, we have,

$$\begin{aligned} V^{(\Omega)}(t+\tau) - V^{(\Omega)}(t) &= \tfrac{1}{\sqrt{\Omega}} S\mathcal{K}^{(\Omega)} - \sqrt{\Omega}\int_t^{t+\tau} \Omega\mathbf{h}(\mathbf{X}^{(\Omega)}(s),\theta)\ \mathrm{d}s + ... \\ &\quad ... + \sqrt{\Omega}\int_t^{t+\tau} \Omega\mathbf{h}(\mathbf{X}^{(\Omega)}(s),\theta)\ \mathrm{d}s - \sqrt{\Omega}\int_t^{t+\tau} A(\phi(s))\ \mathrm{d}s, \\ &= \tfrac{1}{\sqrt{\Omega}} S\mathcal{K}^{(\Omega)} - \sqrt{\Omega}\int_t^{t+\tau} \Omega\mathbf{h}(\mathbf{X}^{(\Omega)}(s),\theta)\ \mathrm{d}s + ... \\ &\quad ... + \sqrt{\Omega}\int_t^{t+\tau} \big(A(\mathbf{X}^{(\Omega)}(s)) - A(\phi(s))\big)\ \mathrm{d}s. \end{aligned}$$

By the central limit theorem the term, $\frac{1}{\sqrt{\Omega}} S\mathcal{K}^{(\Omega)} - \sqrt{\Omega}\int_t^{t+\tau} \Omega\mathbf{h}(\mathbf{X}^{\mathrm{L}}(s),\theta)\ \mathrm{d}s \to \mathcal{Z}^{(\Omega)}$, where,

$$\mathcal{Z}^{(\Omega)} \sim N\bigg(0,\ S\int_t^{t+\tau} \mathbf{h}(\mathbf{X}^{(\Omega)}(s),\theta)\ \mathrm{d}s S^T\bigg). \tag{A.14}$$

Moreover, under the assumptions that there exists a unique solution to the initial value problem (A.5) and that the hazard rates are multinomial (assumptions that are immediately satisfied by a stochastic reaction network), theorem K of Barbour (1974) allows one to rewrite the following,

$$\begin{aligned} \sqrt{\Omega}\int_t^{t+\tau} \big(A(\mathbf{X}^{(\Omega)}(s)) - A(\phi(s))\big)\ \mathrm{d}s &\approx \sqrt{\Omega}\int_t^{t+\tau} \big(\nabla A(\phi(s))(\mathbf{X}^{(\Omega)}(s) - \phi(s))\big)\ \mathrm{d}s \\ &= \int_t^{t+\tau} \big(J(\phi(s))V^{(\Omega)(s)}\big)\ \mathrm{d}s \end{aligned}$$

where $J(\phi(s))$ is the Jacobian, $J_{ij} := \frac{\partial A_j}{\partial \phi_i}$, of the macroscopic ODE. Consequently, taking the limit as $\Omega \to \infty$, and defining $\xi(t) := \lim_{\Omega\to\infty} V^{(\Omega)}(t) = \lim_{\Omega\to\infty}(\sqrt{\Omega}(\mathbf{X}^{(\Omega)}(t) - \phi(t)))$, equation (A.13) becomes,

$$\xi(t+\tau) - \xi(t) = \int_t^{t+\tau} J(\phi(s))\xi(s)\ \mathrm{d}s + \mathcal{Z}, \tag{A.15}$$
$$Z \sim N\bigg(0,\ \int_t^{t+\tau} B(\phi(s))\ \mathrm{d}s\bigg), \tag{A.16}$$

where B is as in (A.11) and comes from equation (A.14).

We therefore arrive at the full specification of the LNA,

$$\mathbf{X}^{\mathrm{L}}(t) = \phi(t) + \Omega^{-1/2}\xi(t), \tag{A.17}$$

where,

$$\frac{\mathrm{d}\phi}{\mathrm{d}t} = A(\phi(t)) \tag{A.18}$$

$$\mathrm{d}\xi = J(\phi(t))\xi(t)\mathrm{d}t + \sqrt{B(\phi(t))}dW_t, \tag{A.19}$$

where dW_t are independent Wiener processes. Equation (A.19) is linear with Itô representation and thus the transition, $\mathbb{P}(\xi(t+\tau)|\xi(t))$, is Gaussian with mean and variance defined by (Komorowski *and others*, 2009),

$$\frac{\mathrm{d}\mu}{\mathrm{d}t} = J(\phi(t))\mu(t) \tag{A.20}$$

$$\frac{\mathrm{d}\Sigma}{\mathrm{d}t} = \Sigma(t)J(\phi(t))^T + J(t)\Sigma(t)^T + B(\phi(t))B(\phi(t))^T. \tag{A.21}$$

Correspondingly, the transition probabilities of the state vector are derived to be (Finkenstädt *and others*, 2013),

$$\mathbb{P}(\mathbf{X}^{\mathrm{L}}(t+\tau)|\mathbf{X}^{\mathrm{L}}(t)) = N(\phi(t) + \Omega^{-1/2}\mu(t+\tau), \Omega^{-1}\Sigma(t+\tau)).$$

The LNA can always be expressed as a linear Gaussian state space model, for simplicity we consider $\mathbf{X}^{\mathrm{L}}(t)$ to be the LNA of a linear stochastic reaction system. In this case, the Jacobian is independent of ϕ and consequently is constant in time so that the state representation takes the form,

$$\mathbf{X}^{\mathrm{L}}(t+\tau) = e^{J\tau}\mathbf{X}^{\mathrm{L}}(t) + (\phi(t+\tau) - e^{J\tau}\phi(t)) + \Omega^{-1/2}\eta(t+\tau), \tag{A.22}$$

$$\eta(t+\tau) \sim N(0, \Sigma(t+\tau))$$

$$\Sigma(t+\tau) = \int_t^{t+\tau} [e^{J(t+\tau-s)}B(s)][e^{J(t+\tau-s)}B(s)]^T \, \mathrm{d}s.$$

As before, ϕ denotes the solution to the macroscopic ODE and J is the associated Jacobian. To be explicit, for the gene transcription model (2.1)-(2.2), $\phi := (\phi_m, \phi_p)^T$, where ϕ_m and ϕ_p solve the following ODE system,

$$\begin{aligned}\frac{\mathrm{d}\phi_m}{\mathrm{d}t} &= \beta(t) - \delta_m \phi_m(t), \\ \frac{\mathrm{d}\phi_p}{\mathrm{d}t} &= \alpha\phi_m(t) - \delta_p\phi_p(t),\end{aligned}$$

and

$$J = \begin{pmatrix} -\delta_m & 0 \\ \alpha & -\delta_p \end{pmatrix}, \qquad B(\phi(t)) = \begin{pmatrix} \sqrt{\beta(t) + \delta_m\phi_m(t)} & 0 \\ 0 & \sqrt{\alpha\phi_m(t) + \delta_p\phi_p(t)} \end{pmatrix}.$$

A.4 *Birth Death Approximation*

Recall that the gene transcription model consists of four reactions,

$$\emptyset \xrightarrow{\beta(t)} M, \qquad M \xrightarrow{\delta_m} \emptyset, \tag{A.23}$$

$$M \xrightarrow{\alpha} M + P, \quad P \xrightarrow{\delta_p} \emptyset. \tag{A.24}$$

This stochastic reaction network models two species of interest, mRNA (M) and protein (P), with corresponding transition density given by $\mathbb{P}(M(t), P(t)|M(0), P(0))$ which satisfies the following differential master equation,

$$\begin{aligned}\frac{\mathrm{d}}{\mathrm{d}t}\mathbb{P}(m,p,t) &= \beta(t)\,\mathbb{P}(m-1,p,t) + \delta_m(m+1)\,\mathbb{P}(m+1,p,t) \\ &\quad + \alpha m\,\mathbb{P}(m,p-1,t) + \delta_p(p+1)\,\mathbb{P}(m,p+1,t) \\ &\quad - (\beta(t) + \delta_m m + \alpha m + \delta_p p)\,\mathbb{P}(m,p,t).\end{aligned} \tag{A.25}$$

$$\mathbb{P}(m,p,0) = \begin{cases} 1 & \text{if} \quad m = m_0 \quad \text{and} \quad p = p_0, \\ 0 & \text{if} \quad m \neq m_0 \quad \text{or} \quad p \neq p_0. \end{cases} \tag{A.26}$$

Here, $\mathbb{P}(m,p,t)$ denotes the transition density $\mathbb{P}(M(t) = m, P(t) = p|M(0) = m_0, P(0) = p_0)$.

To derive an approximation to this transition density, one can construct an approximate

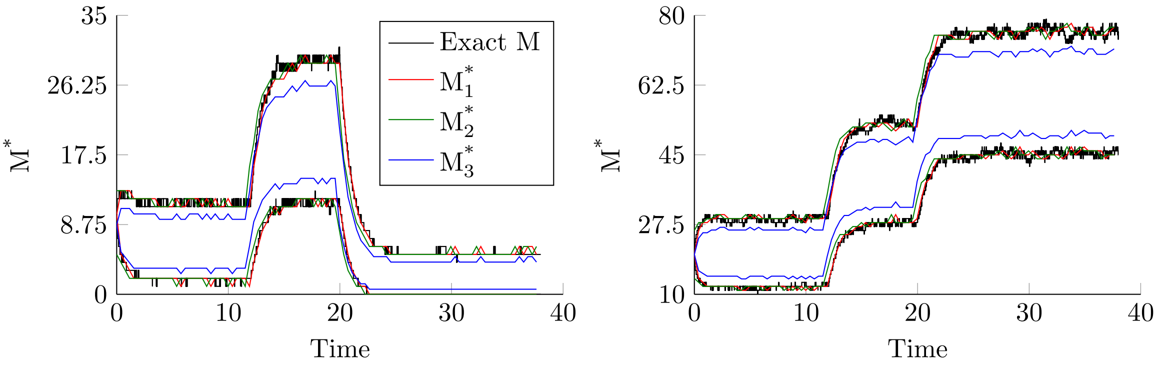

Fig. 1. In each panel, the black envelope is the empirical 95% pointwise simulation interval for the true continuous time mRNA process and the red, green and blue envelopes correspond to the empirical 95% pointwise envelopes for M^* under approximation 1, 2, and 3 respectively. The two panels correspond to different parameter scenarios, corresponding to increasing molecular numbers (left to right).

reaction network that consists of two conditionally independent sub-networks,

$$\emptyset \xrightarrow{\beta(t)} M, \quad M \xrightarrow{\delta_m} \emptyset, \tag{A.27}$$

$$\emptyset \xrightarrow{\alpha M^*(t)} P, \quad P \xrightarrow{\delta_p} \emptyset, \tag{A.28}$$

which corresponds to factorising the joint transition as follows

$$\begin{aligned} \mathbb{P}(M(t), P(t)|M(0), P(0)) &= \mathbb{P}(M(t)|M(0), P(0))\, \mathbb{P}(P(t)|M(t), P(0)) \\ &\approx \mathbb{P}(M(t)|M(0))\, \mathbb{P}(P(t)|M^*(t), P(0)). \end{aligned} \tag{A.29}$$

Note that the exact system will be derived by setting M^* to be the continuous time mRNA process, $M(t)$. We have considered three different definitions of M^*,

1. $M^*(t) := m_0$, the mRNA level at the previous time point. As the distance between observations becomes small, this will converge to the exact process.

2. $M^*(t) := m_t$, the mRNA level at the current time point. Again, as the distance between observations becomes small, this will converge to the exact process.

3. $M^*(t) := \mathrm{E}(M(t)|M(0) = m_0)$, the expected value of the continuous time mRNA process given the previous observation. This approximation will converge to the exact process when

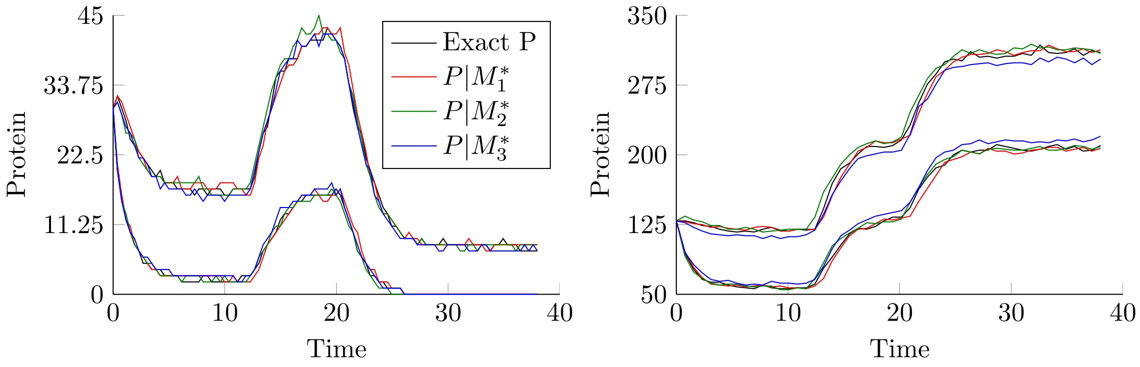

Fig. 2. In each panel, the black envelope is the empirical 95% pointwise simulation interval for the true Protein process and the red, green and blue envelopes correspond to the empirical 95% pointwise envelopes for the approximate Protein process given M^* calculated under approximation 1, 2, and 3 respectively. The two panels correspond to different parameter scenarios, corresponding to increasing molecular numbers (left to right).

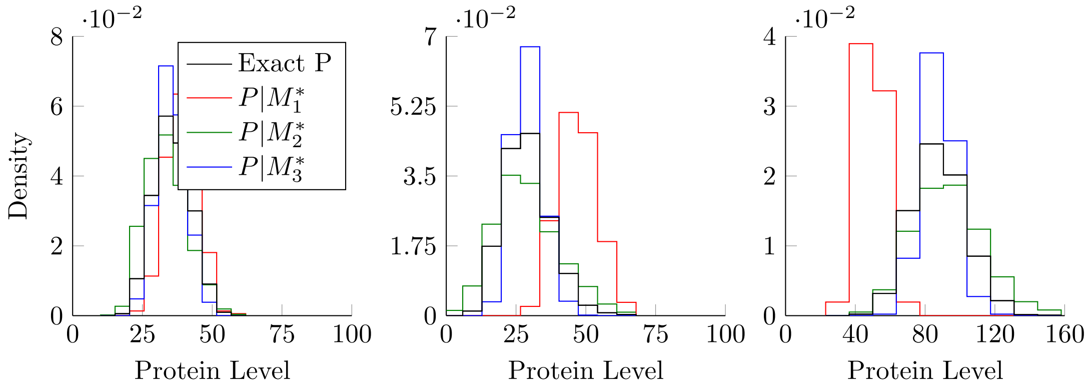

Fig. 3. In each panel, the black histogram is the empirical transition density for the true marginal Protein process and the red, green and blue histograms correspond to the transition densities for the marginal Protein process given M^* calculated under approximation 1, 2, and 3 respectively. Each panel corresponds to a different simulated scenario of different sampling intervals (increasing sampling interval from left to right). In particular, the final panel has a sampling interval large enough to contain a switch in transcription.

either the time between observation becomes small or the intrinsic variability of the mRNA process vanishes.

Note that in all cases, the marginal transition for the mRNA process will remain exact and it is through the protein process that the approximation to the joint transition occurs. Figure 1 shows how each approximation M^* compares to the true underlying process M for 2 different

simulations. Clearly, approximation 3) underestimates the variance in the true process, with only minor differences being observed between the other two approximations. Figure 2 shows the 95 % pointwise simulation intervals for the corresponding Protein population levels under these different simulations for each of the above approximations. Again, it can be seen that under approximation 3), in all scenarios, the variance is underestimated. In comparison, when the sampling interval is small with respect to the speed of the reactions (Figure 2 a)), the difference between approximations 1) and 2) is small. However, as the sampling interval increases, some differences appear particularly about the switch points in transcription. Taking the previous mRNA population as a proxy to the continuous time mRNA process will result in delaying the estimated switch points in transcription, whereas the current mRNA population will accelerate the estimation of switch points. Figure 3, which shows the marginal transition density of the Protein process under each approximation, shows more clearly that as the sampling interval becomes even larger, approximation 2) becomes the more accurate proxy to the true process. Therefore, we restrict our research to using only approximation 2), which we will term the birth-death decomposition (BDD), and note here that approximation 1) may also yield valid inference for systems with a "reasonable" sampling window.

To solve the system (A.27)-(A.28), we note that a birth-death process of the form,

$$\emptyset \xrightarrow{b(t)} X, \quad X \xrightarrow{d(t)} \emptyset, \tag{A.30}$$

has a closed form solution to the corresponding master equation given by,

$$\frac{\mathrm{d}}{\mathrm{d}t}\mathbb{P}(x,t) = b(t)\,\mathbb{P}(x-1,t) + d(t)\,\mathbb{P}(x+1,t) - (b(t)+d(t))\,\mathbb{P}(x,t), \tag{A.31}$$

$$\mathbb{P}(x,0) = \begin{cases} 1 & \text{if } x = x_0 \\ 0 & \text{if } x \neq x_0. \end{cases}$$

Explicitly, letting Z denote the random variable with transition density satisfying (A.31) then,

$$Z = Z_P + Z_B,$$

where $Z_P \sim Pois(\lambda)$ and $Z_B \sim Bin(x_0, \pi)$ and the corresponding parameters satisfy the following system of ODEs,

$$\begin{aligned} \frac{\mathrm{d}\lambda}{\mathrm{d}t} &= b(t) - d(t)\lambda(t), \quad \lambda(0) = 0, \\ \frac{\mathrm{d}\pi}{\mathrm{d}t} &= -d(t)\pi(t), \qquad \pi(0) = 1. \end{aligned}$$

A full derivation of this result is given in Gardiner (1985). This birth-death decomposition or BDD may be further approximated by replacing the Poisson-binomial convolution by a bivariate normal density truncated to the positive real line so that the transition densities are given by,

$$M(t)|M(0) \sim N_T(\lambda^m + m_0\pi^m, \lambda^m + m_0\pi^m(1 - \pi^m)) \tag{A.32}$$

$$P(t)|(M^*(t), P(0)) \sim N_T(\lambda^p + p_0\pi^p, \lambda^p + p_0\pi^p(1 - \pi^p)) \tag{A.33}$$

where N_T indicates the normal density truncated to the positive real line and,

$$\frac{\mathrm{d}\lambda^m}{\mathrm{d}t} = \beta(t) - \delta_m\lambda^m(t), \quad \frac{\mathrm{d}\pi^m}{\mathrm{d}t} = -\delta_m\pi^m(t), \quad \lambda^m(0) = 0, \quad \pi^m(0) = 1. \tag{A.34}$$

$$\frac{\mathrm{d}\lambda^p}{\mathrm{d}t} = \alpha m_0 - \delta_p\lambda^p(t), \quad \frac{\mathrm{d}\pi^p}{\mathrm{d}t} = -\delta_p\pi^p(t), \quad \lambda^p(0) = 0, \quad \pi^p(0) = 1. \tag{A.35}$$

This normal approximation to the BDD is then termed the birth-death approximation of BDA as referred to in the main paper.

B. Inference for state space models

Suppose that we have observations $Y_0, ..., Y_T$ occurring at arbitrary times $(t_0, ..., t_T)$, and let $\mathbf{X}_0, ..., \mathbf{X}_T$ denote the sequence of states $(\mathbf{X}(t_0), ..., \mathbf{X}(t_T))$. Therefore, in the presence of a measurement equation, any of the above approximations to the exact stochastic reaction network can be modelled by a state space model of the form,

$$\mathbf{X}_{t+1} \sim h(\mathbf{x}_{t+1}|\mathbf{x}_t, \theta) \quad Y_t \sim g(y_t|\mathbf{x}_t, \theta). \tag{B.1}$$

Here, h is the transition density and g is the density of the measurement equation. As stated in the main paper, the data likelihood is given by,

$$f(\mathbf{y}|\theta) = \int_{\mathbf{x}} f(\mathbf{y}, \mathbf{x}|\theta) \mathrm{d}\mathbf{x} \tag{B.2}$$

$$= \int_{\mathbf{x}} h(x_0|\theta) g(y_0|x_0, \theta) \prod_{t=1}^{T} h(x_t|x_{t-1}, \theta) g(y_t|x_t, \theta) \mathrm{d}\mathbf{x}. \tag{B.3}$$

B.1 *Kalman Methodology*

Under the LNA with Gaussian measurement error, (B.1) becomes a linear Gaussian state space model and consequently, the likelihood (B.2) can be evaluated explicitly. In this case, equation (B.1) can be expressed in the following matrix form,

$$\mathbf{X}_{t+1} = F_t \mathbf{X}_t + c_t + \eta_t, \tag{B.4}$$

$$Y_t = G_t \mathbf{X}_t + \epsilon_t \tag{B.5}$$

$$\eta_t \sim N(0, \Sigma_t), \quad \epsilon_t \sim N(0, \Sigma_\epsilon)$$

for matrices, $F_t, G_t, \Sigma_t, \Sigma_\epsilon$ that are *independent* of the states $\mathbf{X}_t$. Specifically, the following recursive algorithm (Kalman, 1960) can be used to integrate over the latent states $\mathbf{X}_t$,

1. Assign a prior distribution to the initial latent state, $\mathbf{X}_0 \sim N(a_0, Q_0)$.

Letting $S_0 := G_0 \mathbf{X}_0 G_0^T + \Sigma_\epsilon$, we get the predictive distribution,

$$Y_0 | \mathbf{X}_0 = \mathbf{x}_0 \sim N(G_0 \mathbf{x}_0, S_0). \tag{B.6}$$

2. For time $t = 1, ..., T$,

   (a) Using the state equation (B.4), the predictive distribution for $\mathbf{X}_t | y_{0:t-1}$ becomes $N(b_t, R_t)$, where the predictive equations are given by,

$$b_t = F_{t-1} a_{t-1} + c_{t-1}, \tag{B.7}$$

$$R_t = F_{t-1} Q_{t-1} F_{t-1}^T + \Sigma_{t-1}. \tag{B.8}$$

   (b) Using the observation equation (B.5), we can obtain the predictive distribution for the observations $Y_t | y_{0:t-1}, \mathbf{X}_t = \mathbf{x}_t$, yielding the predictive error,

$$Y_t | \mathbf{X}_t = \mathbf{x}_t \sim N(G_t \mathbf{x}_t, S_t), \tag{B.9}$$

$$S_t = G_t R_{t-1} G_t^T + \Sigma_\epsilon. \tag{B.10}$$

   In addition, the marginal predictive distribution for $Y_t | y_{0:t-1}$, is given by $N(G_t b_t, S_t)$

   (c) Finally, the posterior predictive distribution is given by $\mathbf{X}_t | y_{0:t} \sim N(a_t, Q_t)$, where the updating equations are given by,

$$a_t = b_t + R_t G_t^T R_t^{-1} (y_t - G_t b_t) \tag{B.11}$$

$$Q_t = R_t - R_t G_t^T S_t^{-1} G_t R_t. \tag{B.12}$$

   The above results follow directly from the normality of $Y_t | y_{0:t-1}, \mathbf{X}_t = \mathbf{x}_t$ and $\mathbf{X}_t | y_{0:t-1}$.

The likelihood for the data $\mathbf{y} := y_{0:T}$ is then given by the product of the marginal predictive distributions,

$$\begin{aligned} f(\mathbf{y}|\theta) =& f(y_0|\theta)\prod_{t=1}^{T} f(y_t|y_{0:t-1}) \\ =& \tfrac{1}{(2\pi)^{T/2}|S_0|}\exp\Big(-\tfrac{1}{2}(y_0 - G_0 b_0)^T S_0^{-1}(y_0 - G_0 b_0)\Big)\times ... \\ & ...\times\prod_{t=1}^{T}\tfrac{1}{(2\pi)^{n/2}|S_t|}\exp\Big(-\tfrac{1}{2}(y_t - G_t b_t)^T S_t^{-1}(y_t - G_t b_t)\Big). \end{aligned} \tag{B.13}$$

In order to use the above methodology, one needs to specify a starting value for the recursions, a_0 and Q_0. These starting values may depend upon the parameter vector θ. In particular, Komorowski *and others* (2009) treat a_0 as an additional parameter to be estimated and $Q_0 := \Sigma_{-1}$ is chosen to satisfy the following equation,

$$0 = J_0\Sigma_{-1} + \Sigma_{-1}J_0^T + B_0 B_0^T,$$

where $J_0 := J(\phi(0))$ and $B_0 := B(\phi(0))$. This ensures that the initial covariance matrix is set to be the covariance of the system at time $t = 0$ if the system was initialised at a steady state.

The restarting variant of the LNA (Fearnhead *and others*, 2014) uses the predictive distribution calculated in (B.11) of the Kalman filter, to reset the ODEs, ϕ, used in the transition densities. Specifically, the ODE is recalculated at each time point subject to the condition, $\phi_{t-1} = a_{t-1}$, where ϕ_{t-1} is the ODE evaluated at time point $t-1$. Note that under this Gaussian framework, a_{t-1} is the best linear unbiased predictor of $\mathbf{X}_{t-1}$ and is often denoted by $\hat{\mathbf{X}}_{t-1}$. The restarting LNA method of Fearnhead *and others* (2014) is essential for non-linear systems as it reduces the impact of the initial value. For linear systems, the difference becomes between the restarting and non-restarting methods is reduced, in addition, due to the recursive nature of the restarting method, the implementation can become considerably slower and we consequently focus our attention on the non-restarting version for this (piecewise-linear) application to the gene transcription model of (2.1)-(2.2).

B.2 *Inference for non-linear or non-Gaussian state space models*

When a state space model of the form (B.1) is either non-linear or non-Gaussian, it is often the case that the data likelihood (B.2) cannot be explicitly evaluated. One way in which inference may be performed in this scenario is to target an extended space, $f(\mathbf{x}, \theta|\mathbf{y})$, consisting of both the parameter vector, θ and the latent state variables, $\mathbf{x}$. As stated in the main body of the paper, this posterior can be targeted through the following two step Gibbs procedure,

1. Sample the parameter vector θ from $f(\theta|\mathbf{y}, \mathbf{x})$.
2. Sample the latent states, $\mathbf{x}$, from the filtering density, $f(\mathbf{x}|\mathbf{y}, \theta)$.

B.2.1 *Particle Gibbs.* In this paper we have implemented a particle Gibbs step (Andrieu *and others*, 2010, 2009) in order to perform step 2 of the above Gibbs procedure. This is an extension of the sequential importance sampling (SIS) algorithm of Doucet *and others* (2000), which is outlined below.

Particle filters can be used to sequentially approximate the filtering density, $f(x_t|y_{0:t})$. In particular, the filtering density is approximated by the discrete approximation,

$$f^{N_p}(x_t|y_{0:t}) = \sum_{i=1}^{N_p} w_t^{(i)} \delta_{x_t^{(i)}},$$

where δ_x is a delta function centred at x and $w_t^{(i)}$ are the importance weights. There are two steps needed to obtain a sample $\{x_t^{(i)}, w_t^{(i)}\}$:

1. Sample $x_t^{(i)} \sim q(.|x_{t-1}^{(i)}, y_{0:t})$, where q is the importance density.
2. Compute the importance weights.

Given, the approximate filtering density $f^{N_p}(x_{0:T}|y_{0:T})$, one can obtain a sample of the latent states $\mathbf{x} := x_{0:T}$ as required to calculate the extended likelihood under, for example, the BDA.

The SIS develops from a recursive specification for $f(x_{0:t}|y_{0:t})$, namely,

$$f(x_{0:t+1}|y_{0:t+1}) \propto g(y_{t+1}|x_{t+1})h(x_{t+1}|x_t)f(x_{0:t}|y_{0:t}),$$

where h and g are the state and observation densities as given in the main paper. This means that both the importance density and the weights are defined recursively,

$$\begin{aligned} q(x_{0:t+1}|y_{0:t+1}) &\propto q(x_{t+1}|y_{t+1}, x_t)q(x_{0:t}|y_{0:t}), \\ w_{t+1}^{(i)} &= \frac{f(x_{0:t+1}|y_{0:t+1})}{q(x_{0:t+1}|y_{0:t+1})} \\ &\propto w_t^{(i)} \frac{g(y_{t+1}|x_{t+1})h(x_{t+1}|x_t)}{q(x_{t+1}|y_{t+1}, x_t)}. \end{aligned}$$

In practice, this importance sampling procedure can lead to a *weight degeneracy* problem, where only a small number of samples have a significant weight. To overcome this issue, one can use a resampling procedure where the particle approximation $\{x_t^{(i)}, w_t^{(i)}\}$ is transformed into an equally weighted sample by sampling with replacement. The full SIS algorithm with resampling is given in Algorithm 1.

Algorithm 1. SIS algorithm with resampling

1: At time $t = 0$, sample N_p particles from initial distribution $X_0 \sim N_T(a_0, Q_0)$ to obtain a sample $x_0^{(1)}, ..., x_0^{(N_p)}$. Compute the weights and normalise, where,

$$w_0^{(i)} \propto \frac{g(y_0|x_0^{(i)})h(x_0^{(i)})}{q(x_0^{(i)}|y_0)}.$$

2: For $t = 1, ..., T$,

a) for $i = 1, ..., N_p$,

sample $X_t^{(i)} \sim q(.|x_{0:t-1}^{(i)}, y_{0:t})$ to obtain N_p paths $x_{0:t}^{(1)}, ..., x_{0:t}^{(N_p)}$.

b) Calculate the incremental importance weights,

$$w_t^{(i)} \propto w_{t-1}^{(i)} \frac{g(y_t|x_t^{(i)})h(x_t^{(i)}|x_{t-1}^{(i)})}{q(x_t^{(i)}|x_{0:t-1}^{(i)}, y_{0:t})}.$$

c) Normalise weights,

$$W_t^{(i)} = \frac{w_t^{(i)}}{\sum_{j=1}^{N_p} w_t^{(j)}}.$$

d) Calculate the estimated effective sample size,

$$\hat{N}_{\text{eff}} = 1/\sum_{i=1}^{N_p} \left(W_t^{(i)}\right)^2.$$

If $\hat{N}_{\text{eff}} < \hat{N}_{\text{Thres}}$, resample the paths with weights, $W_t^{(i)}$, and set $W_t^{(i)} = \frac{1}{N_p}$ for $i = 1, ..., N_p$.

3: Sample from $1, ..., N_p$ with weights $W_T^{(1)}, ..., W_T^{(N_p)}$ to obtain a sample of the full latent path $x_{0:T}^{(B)}$ consistent with the data $\mathbf{y}$. Define the ancestral lineage, $B = (B_0, ..., B_T)$ of the sample as the sequence of indices of the particle parents.

A key technicality of the particle filter is the resampling step 2 d) of Algorithm 1. This is used to ensure we can efficiently sample paths compatible with the data, although it may result in a *particle degeneracy* problem. Thus, we need to sample enough particles to ensure that there is a sufficient number of independent paths at the end of the algorithm.

Since the particle filter is embedded within a Gibbs algorithm, one also needs to condition on the latent path used in the previous iteration of the outer MCMC loop. We do this by using a conditional systematic resampling algorithm (Kitagawa, 1996; Andrieu *and others*, 2010). Further technical details of how to embed the SIS within a conditional framework are given in Andrieu *and others* (2010, 2009). The main outline of the algorithm is given in Algorithm 2.

Algorithm 2. Particle Gibbs SIS

Initialisation. Initialise the static parameters, θ and run an SMC method (for example the SIS in Algorithm 1) to obtain a sample $x_{0:T}$ and let B denote its ancestral lineage.

Update. At each iteration of the MCMC, run the following conditional SMC algorithm,

1: At time $t = 0$, for $i \neq B_0$ sample $N_p - 1$ particles from initial distribution $X_0 \sim N(a_0, Q_0)$ to obtain a sample $x_0^{(1)}, ..., x_0^{(B_0)}, ..., x_0^{(N_p)}$. Compute the weights and normalise, where,

$$w_0^{(i)} \propto \frac{g(y_0|x_0^{(i)})h(x_0^{(i)})}{q(x_0^{(i)}|y_0)}.$$

2: For $t = 1, ..., T$,

a) For $i \neq B_t$,

sample $X_t^{(i)} \sim q(.|x_{0:t-1}^{(i)}, y_{0:t})$ to obtain N_p paths $x_{0:t}^{(1)}, ..., x_{0:t}^{(B_{0:t})}, ..., x_{0:t}^{(N_p)}$.

b) Calculate incremental importance weights,

$$w_t^{(i)} \propto w_{t-1}^{(i)} \frac{g(y_t|x_t^{(i)})h(x_t^{(i)}|x_{t-1}^{(i)})}{q(x_t^{(i)}|x_{0:t-1}^{(i)}, y_{0:t})}.$$

c) Normalise weights,

$$W_t^{(i)} = \frac{w_t^{(i)}}{\sum_{j=1}^{N_p} w_t^{(j)}}.$$

d) Calculate the estimated effective sample size,

$$\hat{N}_{\text{eff}} = 1 / \sum_{i=1}^{N_p} \left(W_t^{(i)}\right)^2.$$

If $\hat{N}_{\text{eff}} < \hat{N}_{\text{Thres}}$, resample the paths with weights, $W_t^{(i)}$, conditional on the ancestral lineage B, and set $W_t^{(i)} = \frac{1}{N_p}$ for $i = 1, ..., N_p$.

3: Sample from $1, ..., N_p$ with weights $W_T^{(1)}, ..., W_T^{(N_p)}$ to obtain a sample of the full latent path $x_{0:T}^{(B)}$ consistent with the data $\mathbf{y}$, where B is the updated ancestral lineage.

B.2.2 *Particle Gibbs Implementation of the BDA.* As in the LNA, we treat the initial values of the latent states as additional parameters to be estimated. In addition, in order to implement the particle filter, one needs to define a proposal distribution. This proposal distribution is used to

draw samples of the latent process, x_t, conditional on the observations up to time t and the latent molecular path up to time $t-1$. Since this algorithm is an importance sampler, the proposal distribution, q, will be optimal if it is equal to the filtering density,

$$q(x_t|x_{0:t-1}, y_{0:t}) = f(x_t|x_{0:t-1}, y_{0:t}).$$

If this density is not analytically available, q should otherwise be chosen as a good approximation to $f(x_t|x_{0:t-1}, y_{0:t})$ with slightly heavier tails.

Consider the gene transcription model (A.23) - (A.24) under the BDA. Recall that this decomposition follows the non-linear state space model,

$$Y_t \sim N(\begin{pmatrix} 0 & \kappa \end{pmatrix} \mathbf{X}_t, \sigma_\epsilon^2), \tag{B.14}$$

$$\mathbf{X}_{t+1} \sim N_T(\mu_{t+1}, \sigma_{t+1}^2), \tag{B.15}$$

where $\mathbf{X}_{t+1} := (M_{t+1}, P_{t+1})^T$ and,

$$\mu_{t+1} = \lambda_t + \pi_t \mathbf{X}_t^T,$$

$$\sigma_{t+1}^2 = \lambda_t + \pi_t(1-\pi_t)\mathbf{X}_t^T.$$

The vectors $\lambda_t := (\lambda_t^m, \lambda_t^p)^T$ and $\pi_t := (\pi_t^m, \pi_t^p)^T$ satisfy the ODE system (A.34)-(A.35). Recall that under the BDA, the joint transition density $h(\mathbf{x}_{t+1}|\mathbf{x}_t)$ can be decomposed into,

$$h(m_{t+1}, p_{t+1}|m_t, p_t) = h_m(m_{t+1}|m_t)h_p(p_{t+1}|m_{t+1}, p_t), \tag{B.16}$$

where h_m is the marginal transition for the mRNA process and h_p is the marginal transition density for the Protein process. Consequently, the joint filtering density can also be decomposed as follows,

$$\begin{aligned} f(m_t, p_t|m_{0:t-1}, p_{0:t-1}, y_{0:t}) &= f(p_t|m_{0:t-1}, m_t, p_{0:t-1}, y_{0:t}) \times f(m_t|m_{0:t-1}, p_{0:t-1}, y_{0:t}) \\ &= f(p_t|m_t, p_{t-1}, y_t)h_m(m_t|m_{t-1}), \end{aligned}$$

Letting g denote the observation density, one can propose $x_t := (m_t, p_t)$ in two steps.

1. Propose M_t from the transition density $h_m(m_t|m_{t-1})$ which will have importance weight proportional to 1.

2. Secondly, propose P_t from the proposal $q(p_t|m_t, p_{t-1}, y_t)$ given in (B.17) below.

   In order to construct a reasonable proposal distribution, we note that,

$$f(p_t|m_t, p_{t-1}, y_t) \propto h_p(p_t|p_{t-1}, m_t) g(y_t|p_t),$$

   where h_p is the truncated Gaussian transition density for Protein with mean $\mu_t := \lambda^p + p_0\pi^p$ and variance $\sigma_t^2 := \lambda^p + p_0\pi^p(1-\pi^p)$. Consequently, under the corresponding (non-truncated) normal approximation to h_p, (i.e. $h_p^* \sim N(\mu_t, \sigma_t^2)$),

$$f(p_t|m_t, p_{t-1}, y_t) \overset{\text{approx}}{\propto} h_p^*(p_t|p_{t-1}, m_t) g(y_t|p_t).$$

   Thus enabling the construction of the following proposal proposal distribution for $P_t|P_{t-1}, M_t, Y_t$,

$$P_t|P_{t-1}, M_t, Y_t \sim N(\mu_t^*, {\sigma_t^*}^2) \tag{B.17}$$
$${\sigma_t^*}^2 = \left(\frac{1}{\sigma_t^2} + \frac{\kappa^2}{\sigma_\epsilon^2}\right)^{-1}, \quad \mu_t^* = {\sigma_t^*}^2 \left(\frac{\mu_t}{\sigma_t^2} + \frac{\kappa y_t}{\sigma_\epsilon^2}\right).$$

C. Prior Distributions

Informative Priors. In order to ensure identifiability of the model under both the LNA and BDA methods, informative priors are desirable. In particular, within a single cell imaging framework, one can obtain prior information on the two degradation parameters. These priors are parameterised by log-normal distributions,

$$\log \delta_m \sim N(\mu_{\delta_m}, \sigma_{\delta_m}^2),$$
$$\log \delta_p \sim N(\mu_{\delta_p}, \sigma_{\delta_p}^2).$$

Within simulations, the parameters $\mu_{\delta_m}, \sigma_{\delta_m}, \mu_{\delta_p}, \sigma_{\delta_p}$ were all fixed at the true value. For the application to GFP imaging data, these values were obtained from Finkenstädt *and others* (2013),

where,

$$\mu_{\delta_m} = \log(0.14), \quad \sigma_{\delta_m} = 0.06,$$

$$\mu_{\delta_p} = \log(0.57), \quad \sigma_{\delta_p} = 0.06.$$

Hierarchical Priors. The remaining kinetic and measurement parameters were incorporated within a hierarchy with log-normal priors,

$$\begin{aligned}
\log \alpha &\sim N(\mu_\alpha, \sigma_\alpha^2), \\
\log \beta &\sim \sum_{m=1}^{2} \omega_m N(\mu_{\beta_m}, \sigma_{\beta_m}^2) \\
\log \kappa &\sim N(\mu_\kappa, \sigma_\kappa^2), \\
\log \sigma_\epsilon &\sim N(\mu_\sigma, \sigma_\sigma^2).
\end{aligned}$$

The hyper-parameters were assigned uninformative prior distributions where the mean was given a $N(0, 100^2)$ prior, and the precision was given a $Gamma(1, 0.001)$ prior while the weights of the hierarchical mixture model have a $Dirichlet(2, 2)$ prior. These hyper-parameters are sampled from their conjugate distributions.

In addition, the initial values of the latent states were also incorporated into a hierarchy, with gamma specification, parameterised by the mean and variance,

$$M_0 \sim Gamma(\mu_m, \sigma_m^2),$$

$$P_0 \sim Gamma(\mu_p, \sigma_p^2).$$

The hyper-parameters were again assigned uninformative prior distributions where the mean was given a $N(0, 100^2)$ prior, and the precision was given a $Gamma(1, 0.0001)$ prior. These hyper-parameters are sampled via a Metropolis-Hastings random walk sampler.

Switch Priors. The prior distributions over the switch parameters are chosen to be vague. Specifically, we define a truncated negative binomial distribution over the prior number of switch points

within the data conditional on the number of switches not exceeding some $k_{\max}$. It is assumed that the prior position of switch points is uniform over the entire observation window, $[0, T)$.

$$k \sim NegBin(\mu_k, \sigma_k^2; k_{\max}),$$
$$s_1, ..., s_k | k \sim Unif(0, T).$$

The parameter μ_k, is the prior expected number of switches and should therefore be chosen depending on the data application. For our purposes, this has been fixed at a value of 5 and $k_{\max}$ has been fixed at 20. We have chosen a negative-binomial as opposed to a Poisson prior in order to be less informative about the prior number of switches. In particular, we fix σ_k^2 to be $4\mu_k$.

D. Reversible jump MCMC scheme

At each iteration of the MCMC algorithm, we employ a reversible jump (Green, 1995) step in order to update the log transcriptional profile, $\log \beta(t)$, where,

$$\beta(t) := \beta_j \quad \text{for } t \in [s_j, s_{j+1}).$$

A reversible jump method is used in order to sample across the different model dimensions, corresponding to the number, k, of switch times within the transcriptional profile. This is implemented according to a similar specification as in Jenkins *and others* (2013). At each iteration of the reversible jump step, we allow one of the three following possible moves.

1. **Propose the addition** of a switch with probability b_k.

   A new switch, s^* is proposed uniformly on $[0, T]$. Suppose $s^* \in [s_j, s_{j+1})$, then new values for the transcription rates are required on this interval. In particular, the new rates are defined as a perturbation of the old rates and since we are targeting the log-parameters, this is done by first drawing u uniformly on $[0, 1]$, and setting the new rates β_j^*, β_{j+1}^* so

that the following set of equations are satisfied,

$$\log \beta^*_{j+1} = \log \beta_j + u$$
$$\log \beta^*_j = \log \beta_j - u,$$

where β_j was the original transcription rate over the interval $[s_j, s_{j+1})$. Since the new rates have been proposed as a transformation of the old rates, $\log \beta_j$ and the random variable u, the corresponding Jacobian is given by,

$$J := \begin{vmatrix} \frac{\partial \beta^*_{j+1}}{\partial \beta_j} & \frac{\partial \beta^*_{j+1}}{\partial u} \\ \frac{\partial \beta^*_j}{\partial \beta_j} & \frac{\partial \beta^*_j}{\partial u} \end{vmatrix} = 2.$$

2. **Propose the deletion** of a switch with probability d_k.

   A switch is proposed uniformly from $\{s_1, ...s_k\}$ for deletion. Suppose s_j is the candidate for deletion, then the new transcription rate, β^*, over $[s_{j-1}, s_{j+1})$ will be chosen so that,

$$\log \beta^* = (\log \beta_{j-1} + \log \beta_j)/2.$$

   Associated with this transformation is the inverse Jacobian,

$$J^{-1} := \begin{vmatrix} \frac{\partial \beta^*_{j+1}}{\partial \beta_j} & \frac{\partial \beta^*_{j+1}}{\partial u} \\ \frac{\partial \beta^*_j}{\partial \beta_j} & \frac{\partial \beta^*_j}{\partial u} \end{vmatrix}^{-1} = 1/2.$$

3. **Propose to move** a switch with probability $1 - b_k - d_k$.

   A candidate switch for moving is proposed uniformly from $\{s_1, ...s_k\}$, say s_j. The new placement s^*_j is proposed uniformly on the interval $[s_{j-1}, s_{j+1})$. Since there is no transformation of the rate variables associated with this move, the Jacobian is equal to 1.

As in Green (1995), we let $b_k = c \min(1, f(k+1)/f(k))$, $d_k = c \min(1, f(k-1)/f(k))$, where c is some constant (throughout our implementation, this has been fixed at 0.4) and $f(k)$ is the prior density for k switches. The proposed transcriptional profile obtained from performing one of a), b) or c) is then accepted with probability,

$$\alpha = \min(1, \text{Likelihood Ratio} \times \text{Prior Ratio} \times \text{Proposal ratio} \times \text{Jacobian}). \qquad \text{(D.1)}$$

Note that the prior ratio is comprised of the ratio of the priors over, a) the number of switches, b) the position of switches and c) the transcriptional rates. We note that conditional on k switches, the prior assumption that switches occur uniformly corresponds to the distribution of consecutive switches, $(s_{j+1} - s_j)/T$ following a $Beta(2, 2k)$ distribution (Boys and Giles, 2007).

E. Conjugate Update of the Hierarchical Hyper-parameters

As specified in the main paper, the kinetic and measurement hyper parameters of the hierarchical model can be updated by sampling from the full conditional distributions. To be explicit, we consider the example of translation rates $\alpha := (\alpha^{(1)}, ..., \alpha^{(N)})$ for all cells $1, ..., N$. It is assumed that,

$$\log \alpha \sim N(\mu_\alpha, \sigma_\alpha^2), \tag{E.1}$$

with hyper-prior distributions given by,

$$\mu_\alpha | \sigma_\alpha^2 \sim N(m, (\tfrac{\sigma_\alpha}{t})^2), \quad \sigma_\alpha^{-2} \sim Gamma(a, b). \tag{E.2}$$

Thus, given observations α, the hyper-parameters μ_α and σ_α^2 can be drawn from the following full conditional distribution,

$$\mu_\alpha | \sigma_\alpha^2, \alpha \sim N(m^*, (\tfrac{\sigma_\alpha}{t^*})^2), \quad \sigma_\alpha^{-2} | \alpha \sim Gamma(a^*, b^*), \tag{E.3}$$

where,

$$m^* = (t^{-1} m + N\overline{\alpha}_L)/(t^{-1} + N), \quad 1/t^* = t^{-1} + N.$$

$$a^* = a + N/2, \quad b^* = b + \tfrac{1}{2}(N s_{\alpha L}^2 + (t^{-1} N (\overline{\alpha}_L - m)^2)/(t^{-1} + N),$$

where $\overline{\alpha}_L := \frac{1}{N} \sum \log \alpha$ and $s_{\alpha L}^2 := \frac{1}{N-1} \sum (\log \alpha - \overline{\alpha}_L)^2$. In exactly the same way, the hyper parameters $\mu_\kappa, \sigma_\kappa, \mu_{\sigma_\epsilon}, \sigma_{\sigma_\epsilon}$ can be updated. It has been assumed throughout that all hyper-prior

distributions have the following parameters,

$$m = 0, \quad t^{-1} = 100,$$

$$a = 1, \quad b = 1/1000.$$

In order to update the hyper parameters of the mixture prior for transcription rates, $\beta := (\beta^{(1)}, ..., \beta^{(N)})$ where $\beta^{(i)} := (\beta_0^{(i)}, ..., \beta_K^{(i)})$ is the vector of all rates for all cells $i = 1, ..., N$, we introduce some additional notation. Recall that the hyper-distribution is given by,

$$\log \beta \sim \sum_{v=1}^{V} w_{\beta_v} N(\mu_{\beta_v}, \sigma^2_{\beta_v}). \tag{E.4}$$

Following the derivation given in McLachlan and Peel (2004), let f_v be the density corresponding to component v and let $\zeta = (\zeta_1, ..., \zeta_N)$ be the vector of indicator values such that,

$$\zeta_{iv} = \begin{cases} 1 & \text{if the } i\text{th observation is drawn from } f_v \\ 0 & \text{otherwise.} \end{cases}$$

Given these indicator variables, inference can be performed by a series of Gibbs steps as the likelihood can now be written in the following form,

$$f(\beta, \zeta | \mathbf{w}_\beta, \mu_\beta, \sigma^2_\beta) = f(\zeta | \mathbf{w}_\beta) f(\beta | \zeta, \mu_\beta, \sigma^2_\beta) \tag{E.5}$$

$$= \prod_{i=1}^{N} \prod_{v=1}^{V} \left(w_{\beta_v} f(\beta^{(i)} | \mu_{\beta_v}, \sigma^2_{\beta_v}) \right)^{\zeta_{iv}}. \tag{E.6}$$

With conjugate priors given by,

$$\mathbf{w}_\beta \sim \textit{Dirichlet}(c_1, ..., c_V)$$

$$\zeta | \mathbf{w}_\beta \sim \textit{Multinomial}(1, \mathbf{w}_\beta)$$

$$\mu_{\beta_1}, ..., \mu_{\beta_V} \sim N(m, t)$$

$$\sigma^{-2}_{\beta_1}, ..., \sigma^{-2}_{\beta_V} \sim \textit{Gamma}(a, b).$$

Consequently, in order to update the hyper-parameters, $\mu_\beta, \sigma^2_\beta$ and $\mathbf{w}_\beta$, conditional on the observations $\log \beta$, sample from each of the following full conditional distributions,

1. $\mathbf{w}_\beta | \log\beta \sim Dirichlet(c_1^*, ..., c_V^*)$,

   where $c_v^* = \sum_{i=1}^N \zeta_{iv} + c_v$ for $v = 1, ..., V$.

2. $\zeta | \mathbf{w}_\beta, \log\beta \sim Multinomial(1, \mathbf{w}_\beta^*)$,

   where $w_\beta^* \propto w_\beta f(\log\beta | \mu_{\beta_v}, \sigma_{\beta_v}^2)$.

3. For $v = 1, ..., V$ sample

   (a) $\mu_{\beta_v} | \log\beta \sim N(m^*, t^*)$,

   where, $m^* = (t^{-1}m + N_v\overline{\beta}_L)/(t^{-1} + N_v)$, and $1/t^* = t^{-1} + N_v$, with $N_v := \sum_{i=1}^N \zeta_{iv}$, $\overline{\beta}_L := \frac{1}{N_v}\sum_i \log\beta \times \zeta_{iv}$.

   (b) $\sigma_{\beta_v}^{-2} \sim Gamma(a^*, b^*)$,

   where, $a^* = a + N_v/2$, $b^* = b + \frac{1}{2}(N_v s_{\beta L}^2 + (t^{-1}N_v(\overline{\beta}_v - m)^2)/(t^{-1} + N_v)$ and $s_{\beta L}^2 := \frac{1}{N_v - 1}\sum_i (\log\beta - \overline{\beta}_L)^2 \times \zeta_{iv}$.

F. Simulation Study

We consider 3 scenarios of different parameter choices relating to 3 different underlying population levels where each dataset contains 15 time series each measured over 50 hours with 100 discrete measurements. Within each scenario, time series are simulated with a variety of switching regimes and for each scenario we performed 10 simulations and applied both the BDA and LNA models.

Scenario 1 is simulated from the parameter set: $\log\delta_m \sim N(\log(0.4), 0.02)$, $\log\delta_p \sim N(\log(0.7), 0.02)$, $\log\beta \sim N(\log(8), 0.3)$, $\log\alpha \sim N(\log(4), 0.05)$, $\log\kappa \sim N(\log(2), 0.05)$, $\log\sigma_\epsilon \sim N(\log(4), 0.2)$. For this scenario, the average mRNA level will be approximately 20 and average Protein level will be approximately 115. The transcriptional rates are simulated from a single distribution with an average of 2 switches in each time series.

Scenario 2 is simulated from the parameter set: $\log\delta_m \sim N(\log(0.4), 0.02)$, $\log\delta_p \sim N(\log(0.7), 0.02)$,

$\log\beta \sim 0.5{*}N(\log(2), 0.2){+}0.5{*}N(\log(10), 0.1)$, $\log\alpha \sim N(\log(4), 0.05)$, $\log\kappa \sim N(\log(2), 0.05)$, $\log\sigma_\epsilon \sim N(\log(4), 0.2)$. For this scenario, the average mRNA level will be approximately 15 and average Protein level will be approximately 85. The transcriptional rates are simulated from a bimodal distribution with an average of 2 switches in each time series.

Scenario 3 is simulated from the parameter set: $\log\delta_m \sim N(\log(0.4), 0.02)$, $\log\delta_p \sim N(\log(0.7), 0.02)$, $\log\beta \sim 0.5{*}N(\log(2), 0.2){+}0.5{*}N(\log(4), 0.1)$, $\log\alpha \sim N(\log(1), 0.05)$, $\log\kappa \sim N(\log(4), 0.05)$, $\log\sigma_\epsilon \sim N(\log(5), 0.2)$. For this scenario, the average mRNA level will be approximately 8 and average Protein level will be approximately 11. The transcriptional rates are simulated from a bimodal distribution with an average of 2 switches in each time series.

F.1 *Example*

We present here an example from one simulation under Scenario 3. The 15 simulated time series are shown in Figure 4, where a) gives the simulated transcriptional profiles, b) the unobserved mRNA levels, c) the unobserved protein levels and d) the observed measurements. Specifically, we present the results from running the LNA and the BDA with κ fixed at the true value.

Under the LNA, the MCMC algorithm for this simulation took 400,000 iterations compared to the BDA version which took 1,500,000 iterations to sufficiently explore the posterior. The corresponding trace plots of the thinned Markov chains (every 10 iterations) after an initial burn-in period for the hyper-parameters are shown in Figure 5 for the LNA and Figure 6 for the BDA. Figures 7 and 8 give the trace plots of the thinned Markov chains for the individual parameters of one randomly selected time series from Figure 4 under the LNA and BDA respectively. These thinned Markov chains have been used to obtain an estimate of the marginal posterior distributions, shown in Figures 9-10 (LNA) and Figures 11-12 (BDA). It can be seen that the true values all lie well within the estimated posterior densities.

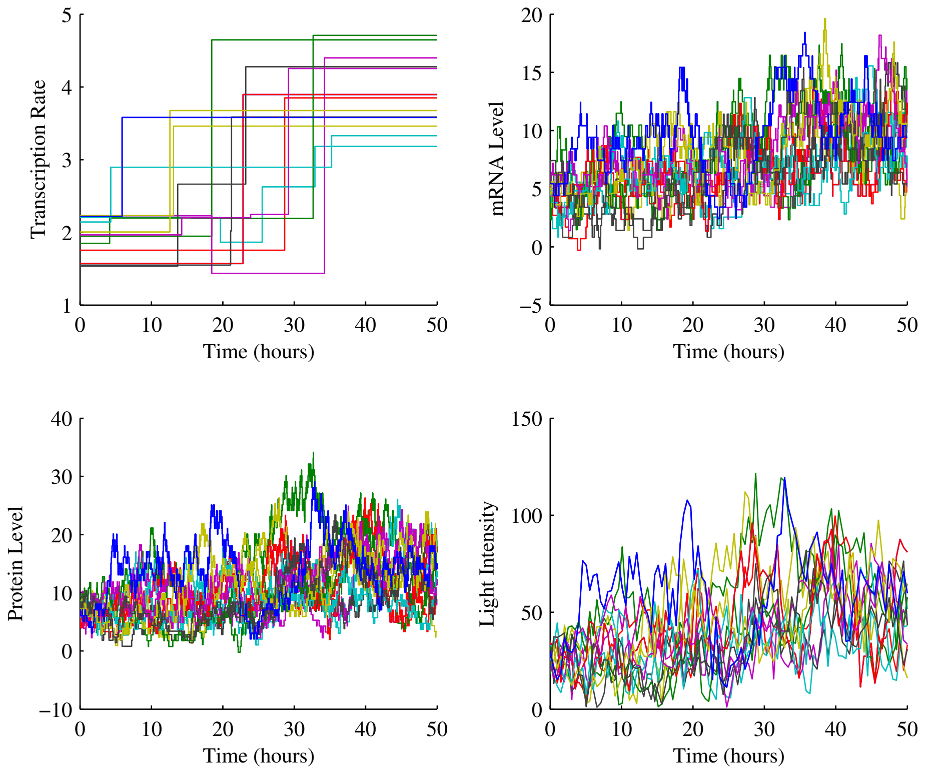

Fig. 4. 15 simulated time series from a single hierarchical distribution. a) gives the simulated transcriptional profiles, b) the corresponding continuous time mRNA process, c) the continuous time protein process and d) the observed measurements.

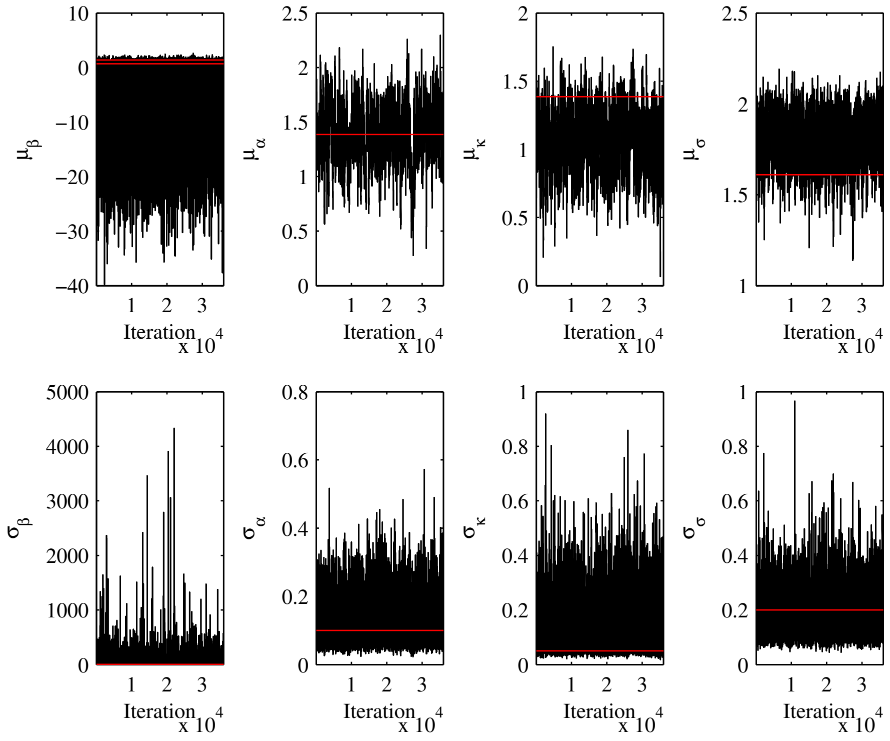

Fig. 5. Trace plots of the thinned Markov chains for each of the hyper parameters calculated under the LNA. Red line indicates the true value.

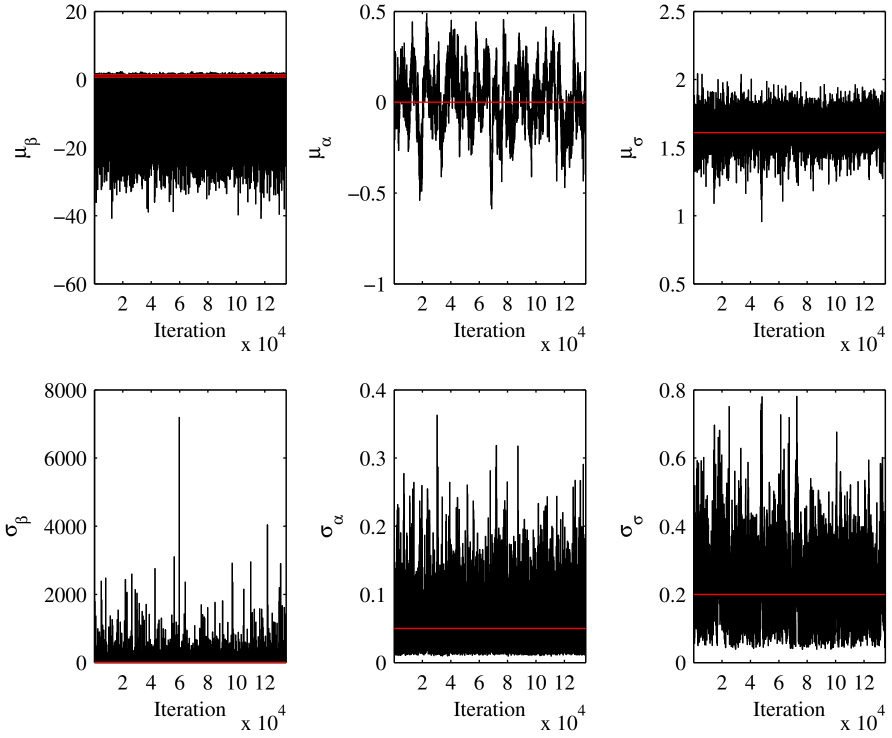

Fig. 6. Trace plots of the thinned Markov chains for each of the hyper parameters calculated under the BDA with red line indicating the true parameter value.

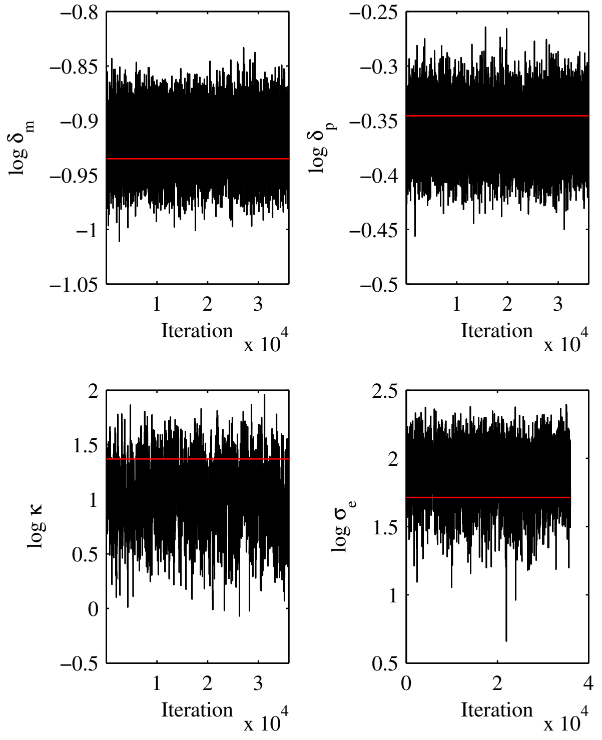

Fig. 7. Trace plots of the thinned Markov chains for each of the individual parameters for a single time series calculated under the LNA with red line indicating the true parameter value.

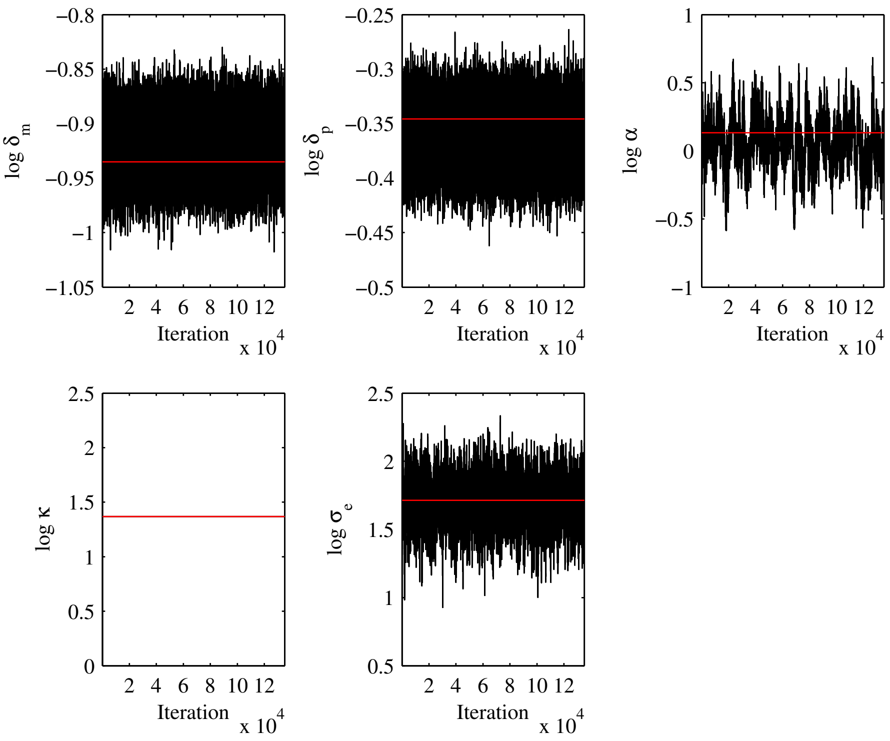

Fig. 8. Trace plots of the thinned Markov chains for each of the individual parameters for a single time series calculated under the BDA with red line indicating the true parameter value.

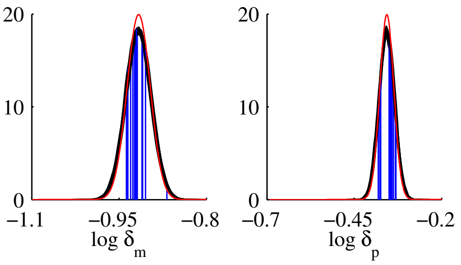

Fig. 9. Posterior densities for the two degradation parameters, calculated under the LNA. True values are shown in blue and prior densities shown in red.

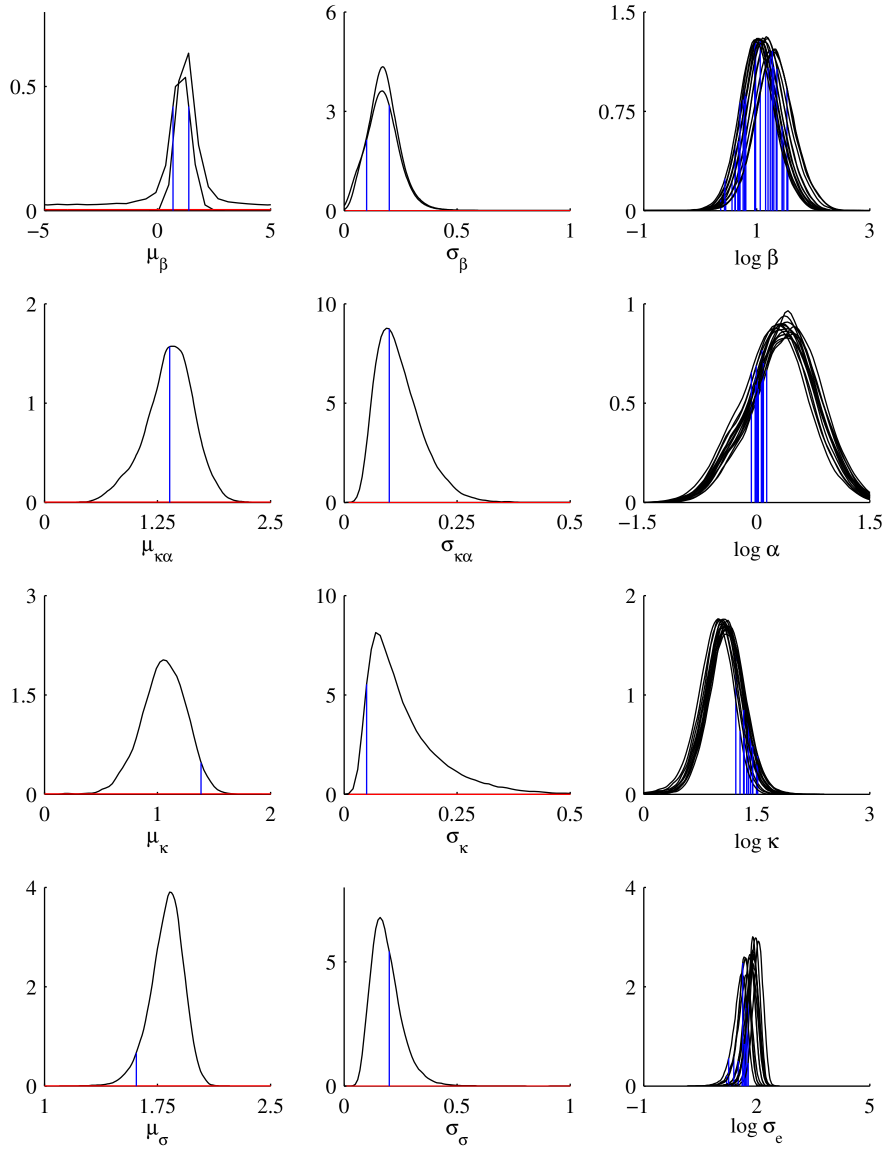

Fig. 10. Posterior densities for the hierarchical parameters, calculated under the LNA. True values are shown in blue and prior densities shown in red.

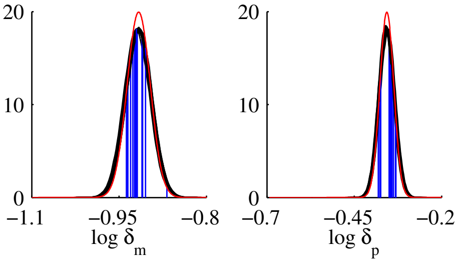

Fig. 11. Posterior densities for the two degradation parameters, calculated under the BDA. True values are shown in blue and prior densities shown in red.

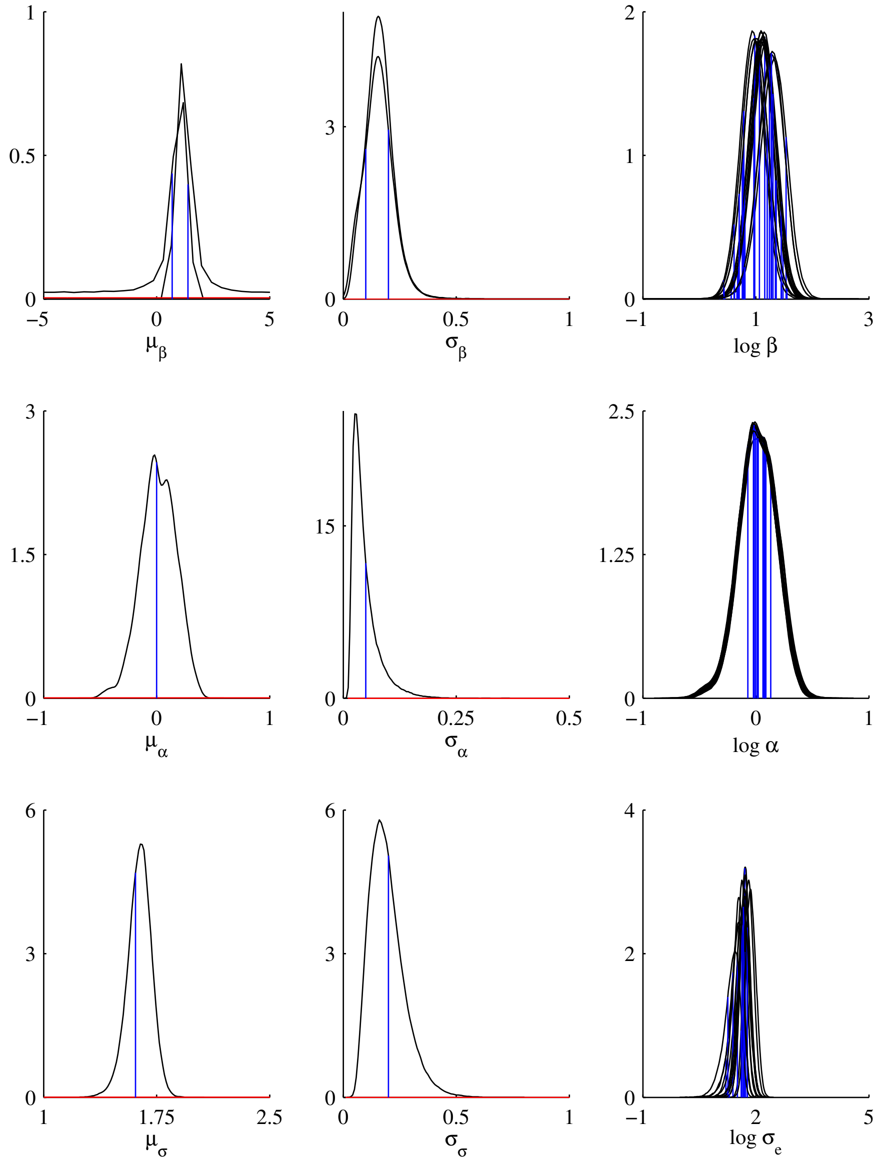

Fig. 12. Posterior densities for the hierarchical parameters, calculated under the BDA. True values are shown in blue and prior densities shown in red.

F.2 *Results*

There are many ways in which one can extract the posterior transcriptional profiles from the reversible jump output. For the purpose of this simulation study, we have only extracted the marginal switch profiles as in Jenkins *and others* (2013). This is achieved by fitting a Gaussian mixture model to the local maxima of the posterior density for switch times. Although this has worked well in simulations, other methods may be applied and in particular, it should be noted that by summarising through the marginal distribution of switch times, we are averaging over the models sampled through the reversible jump methodology. As such, when applying to data, it is recommended that a more detailed analysis of the posterior switch times should be performed.

Figure 13 shows the mean square errors (MSE) of the kinetic parameters calculated at the posterior median values for each of the 3 simulation scenarios. We compare 5 different methods for inferring these parameters:

1. LNA,
2. LNA with κ fixed at the truth,
3. LNA with κ fixed at the LNA posterior median (obtained from method 1)),
4. BDA with κ fixed at the truth,
5. BDA with κ fixed at the LNA posterior median (obtained from method 1)).

Since the BDA cannot reliably estimate the scaling parameter, κ, it needs to be fixed *a priori*. In general, one may not know the value of κ, which motivates methods 3) and 5). A possible alternative to fixing κ would be to run the algorithm over a grid of “reasonable estimates” for κ and perform model selection.

From Figure 13 it can be seen that in some scenarios, the BDA provides a more accurate estimate for the transcription rate, β, and the translation rate, α, regardless of whether the LNA

is calculated with κ fixed at the truth. Interestingly, the LNA does reliably estimate the product $\alpha\beta$ and implies the BDA is better able to distinguish between these two parameters. Moreover, Figure 14 shows the width of the 50% credible intervals under each of the different methods and in general, the BDA tends to give narrower intervals. The main advantage of the LNA is its computational efficiency and furthermore in practice one would be required to run the LNA to first obtain an estimate of κ before running the BDA methodology. These results show that one can use the BDA to further refine the LNA estimates of the kinetic parameters which themselves give reasonable accuracy in reasonable computational run time.

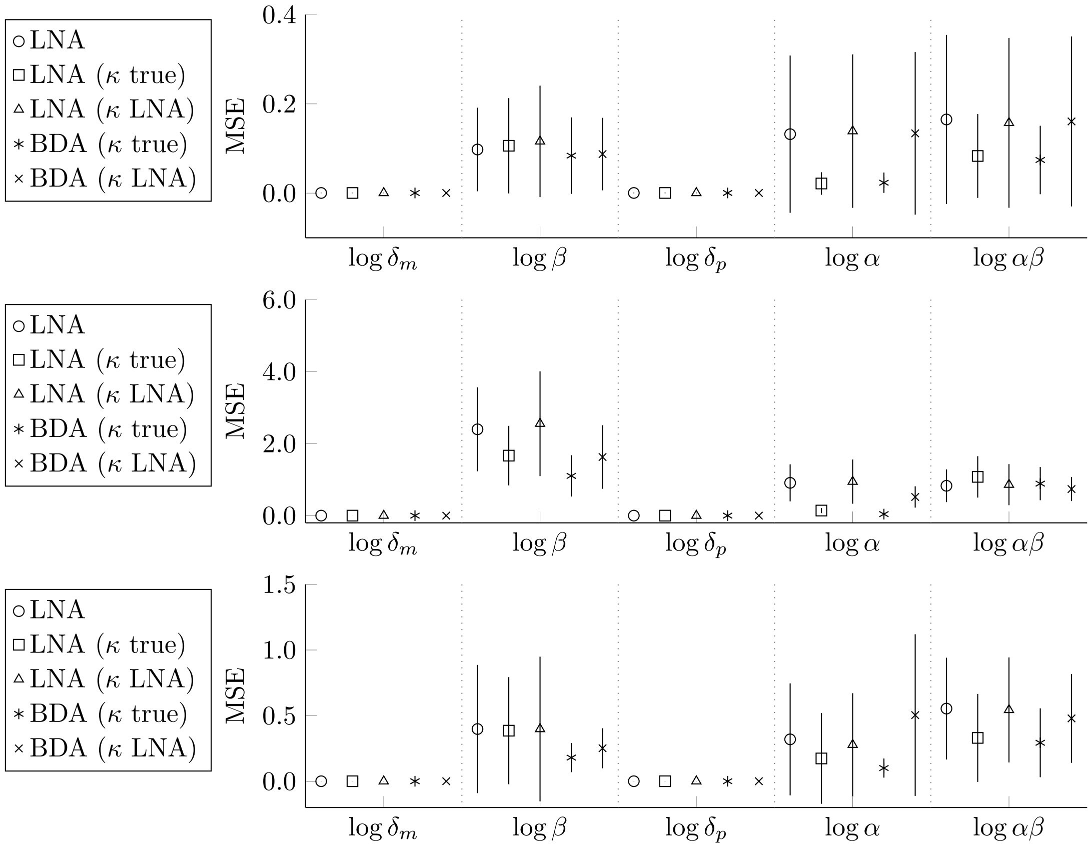

Fig. 13. The mean square error for each estimated parameter calculated under the LNA (circle), the LNA with κ fixed at the truth (square), the LNA with κ fixed at the posterior median of the LNA (triangle), the BDA with κ fixed at the truth (square) and the BDA with κ fixed at the posterior median of the LNA (cross). Each panel corresponds to a different simulation scenario, with Scenario 1 shown in the top panel, Scenario 2 in the middle pan ale and Scenario 3 in the bottom panel. For each scenario, there are 10 different simulations containing 15 individual time series. The MSE is therefore calculated from 150 different estimates of the posterior median. The vertical lines are centred at the mean square error with length given by two standard deviations of the square error for each parameter.

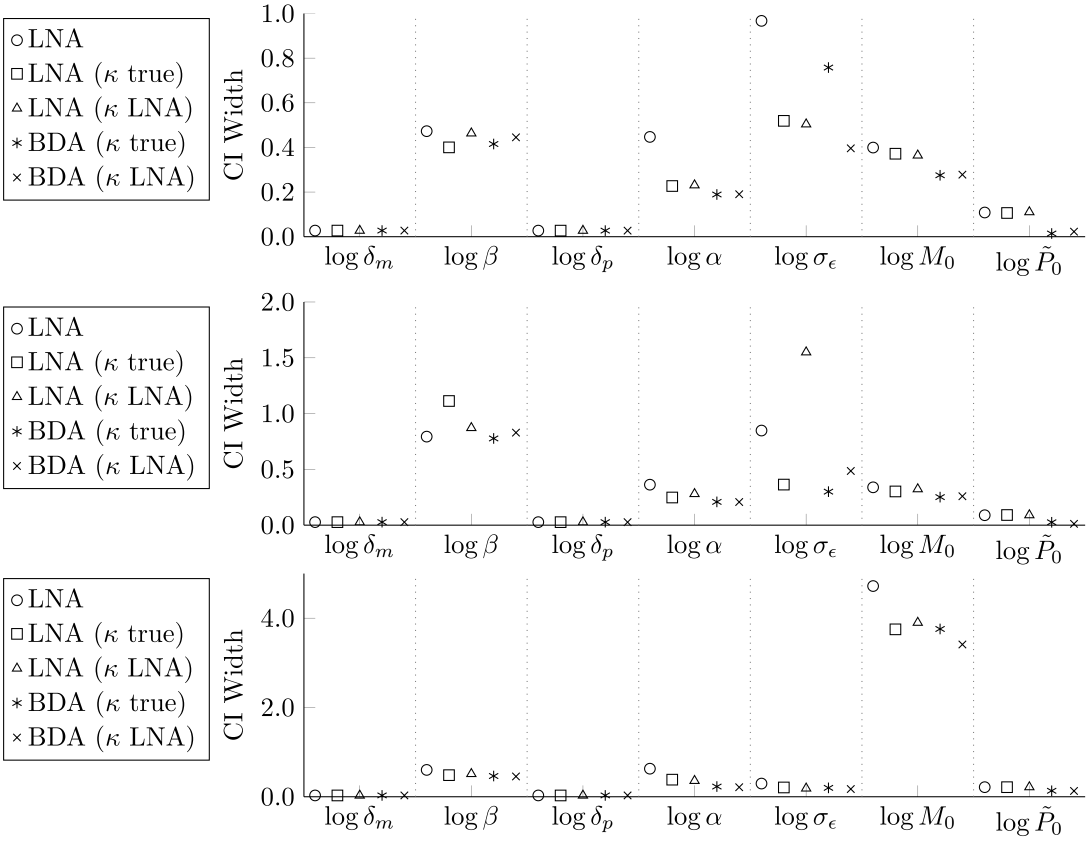

Fig. 14. The width of the 50% credible interval calculated under the LNA (circle), the LNA with κ fixed at the truth (square), the LNA with κ fixed at the posterior median of the LNA (triangle), the BDA with κ fixed at the truth (square) and the BDA with κ fixed at the posterior median of the LNA (cross). Each panel corresponds to a different simulation Scenario, with Scenario 1 shown in the top panel, Scenario 2 in the middle pan ale and Scenario 3 in the bottom panel.

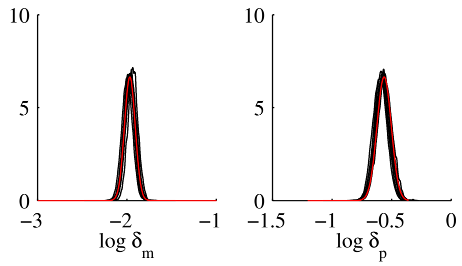

Fig. 15. Posterior densities for the two degradation parameters of the immature dataset, calculated under the LNA. Prior densities are shown in red.

G. Data Application

Provided in Figures 15-22 are the posterior densities obtained from running the LNA and BDA methodologies to the two datasets shown in Figure 2 of the main paper.

Figures 15 and 16 Obtained from applying the LNA to the immature data shown in Figures 2 a) of the main paper.

Figures 17 and 18 Obtained from applying the BDA with κ fixed at the LNA posterior median to the immature data.

Figures 19 and 20 Obtained from applying the LNA to the mature data shown in Figure 2 b) of the main paper.

Figures 21 and 22 Obtained from applying the BDA with κ fixed at the LNA posterior median to the mature data.

In all four cases, the importance of the prior information regarding the degradation parameters, δ_m and δ_p, can be seen. Specifically the posteriors for these parameters are often indistinguishable from the informative prior densities.

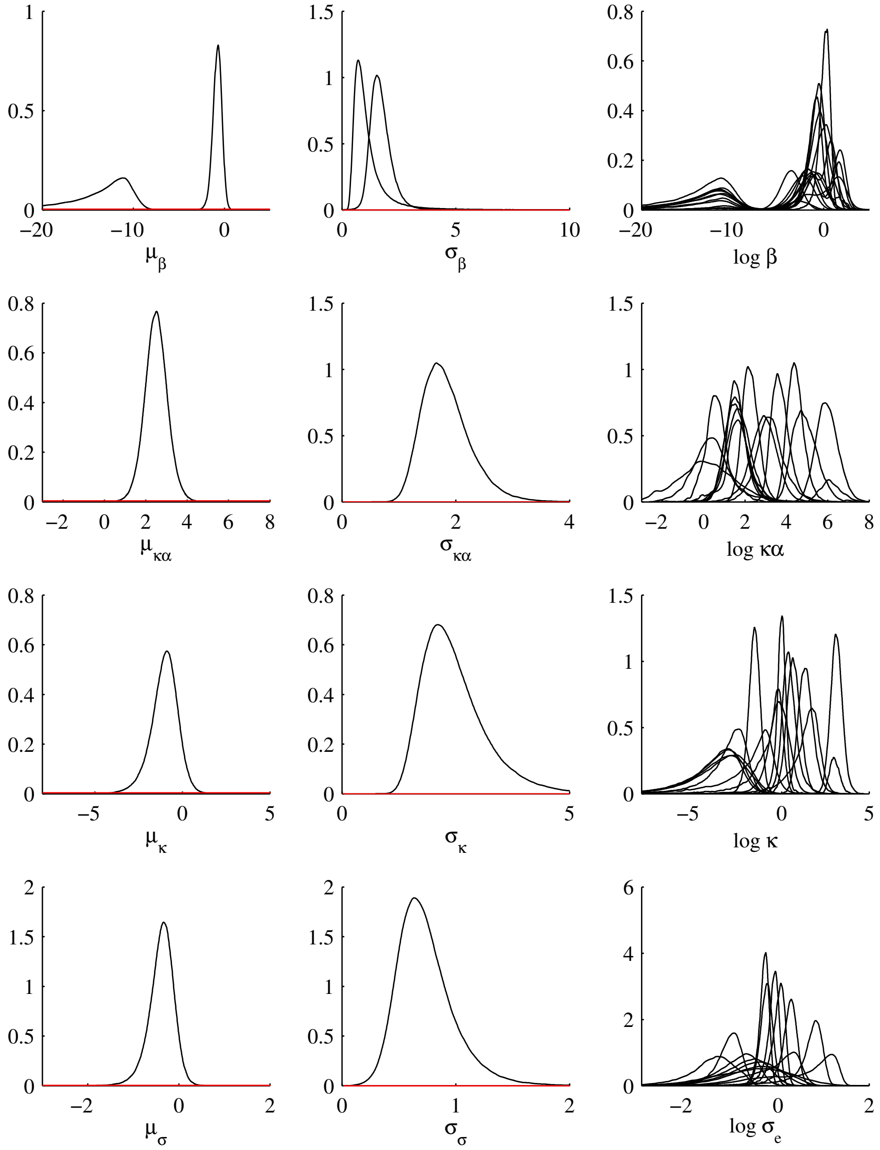

Fig. 16. Posterior densities for the hierarchical parameters of the immature dataset, calculated under the LNA. Prior densities are shown in red.

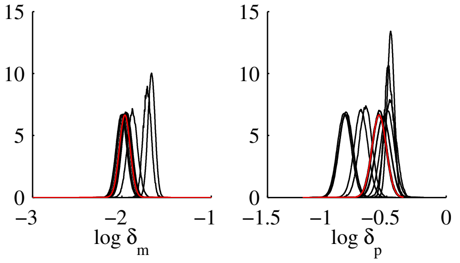

Fig. 17. Posterior densities for the two degradation parameters of the immature dataset, calculated under the BDA. Prior densities are shown in red.

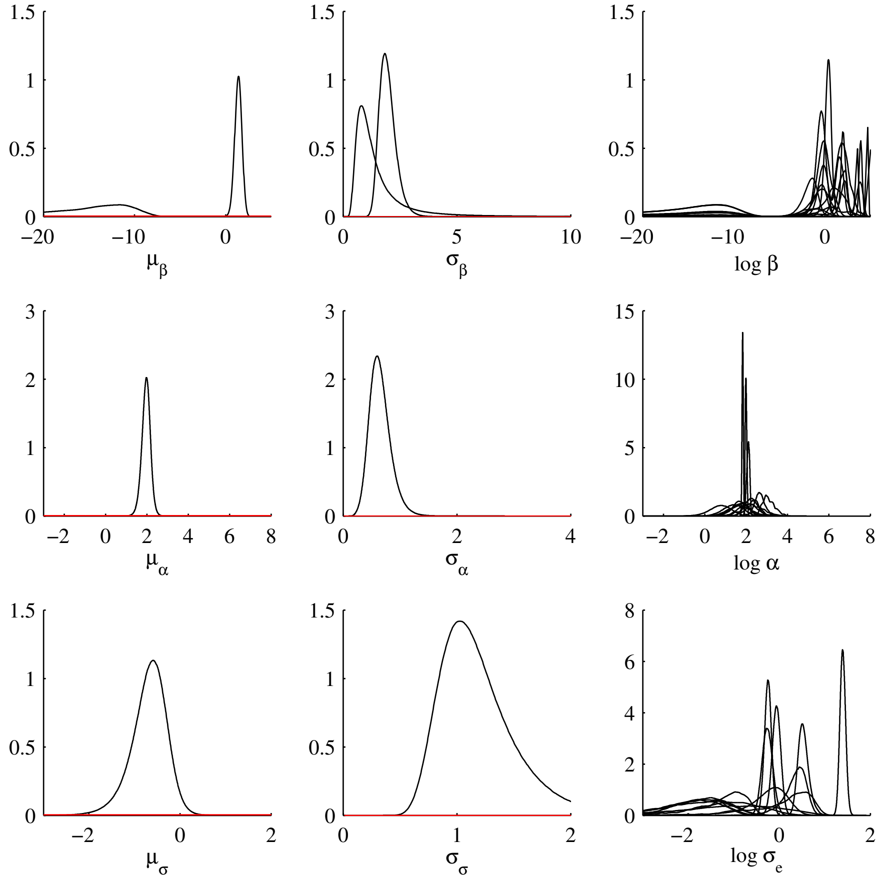

Fig. 18. Posterior densities for the hierarchical parameters of the immature dataset, calculated under the BDA. Prior densities are shown in red.

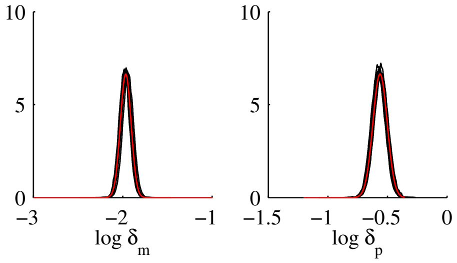

Fig. 19. Posterior densities for the two degradation parameters of the mature dataset, calculated under the LNA. Prior densities are shown in red.

In order to assess how well each of the approximations fit the data, we look at the following recursive residuals,

$$r_t = \frac{y_t - \mathrm{E}(Y_t|y_{1:t-1})}{\sqrt{\mathrm{Var}(Y_t|y_{1:t-1})}}, \quad \text{for} \quad t = 1, ..., T, \tag{G.1}$$

where $Y_t|y^{1:t-1}$ is the one-step ahead predictive distribution. The latter is computed as part of the Kalman filter for the LNA, while under the BDA the moments of the predictive density can be represented by the weighted sample, $\mathrm{E}(h(X_t)|y_{1:t-1}) = \sum_{i=1}^{N_p} w_i h(x_t^{(i)}) / \sum_{i=1}^{N_p} w_i$, for any function h, weights $w_1, ..., w_{N_p}$ and samples $x_t^{(1)}, ..., x_t^{(N_p)}$. Therefore, it is straightforward to extract the recursive residuals for both models. Under a state space formulation the residuals in (G.1) will be i.i.d. with mean zero and variance one if the model fits the data. Moreover, if the state space formulation is Gaussian, the residuals will also be Gaussian. Figure 23 shows the residuals of the LNA and BDA models applied to the time series shown in Figure 9 of the main paper. The residuals were computed at the posterior median of all parameter values and despite the differences in the estimated transcriptional profiles, we see that in both cases, they satisfy all assumptions. This residual analysis was performed on all cells to confirm uncorrelated Gaussian residuals indicating that the stochastic switch model under both the LNA and BDA fits the data well.

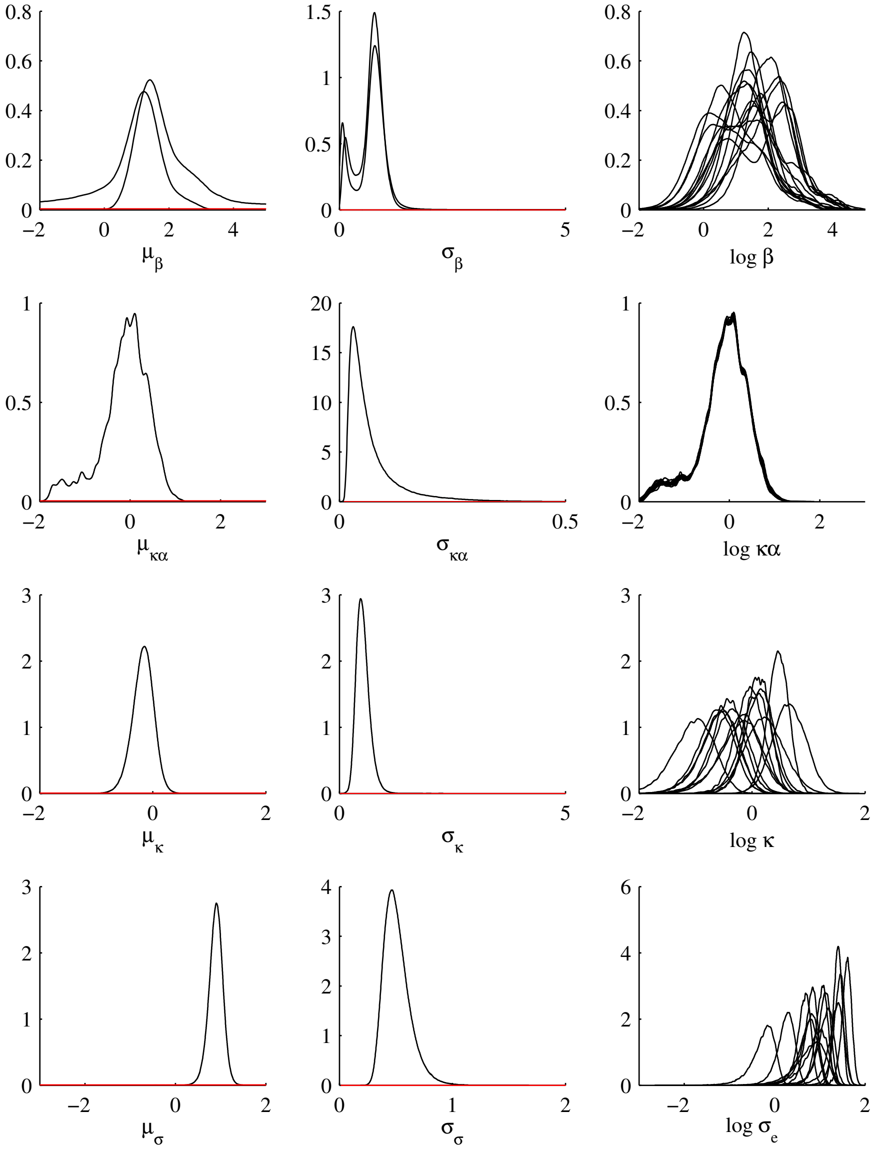

Fig. 20. Posterior densities for the hierarchical parameters of the mature dataset, calculated under the LNA. Prior densities are shown in red.

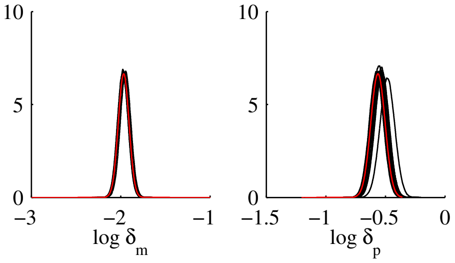

Fig. 21. Posterior densities for the two degradation parameters of the mature dataset, calculated under the BDA. Prior densities are shown in red.

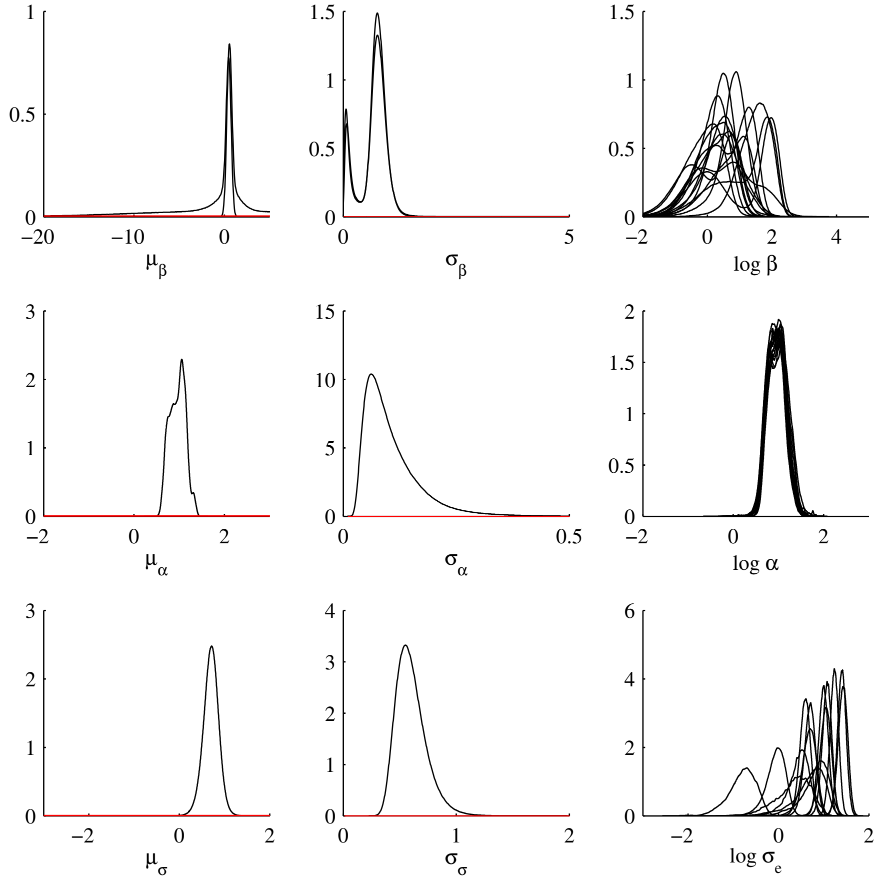

Fig. 22. Posterior densities for the hierarchical parameters of the mature dataset, calculated under the BDA. Prior densities are shown in red.

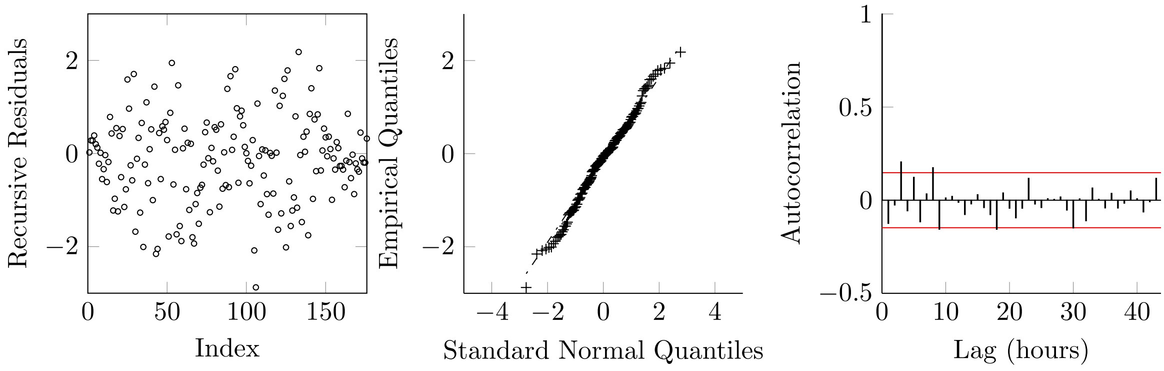

(a)

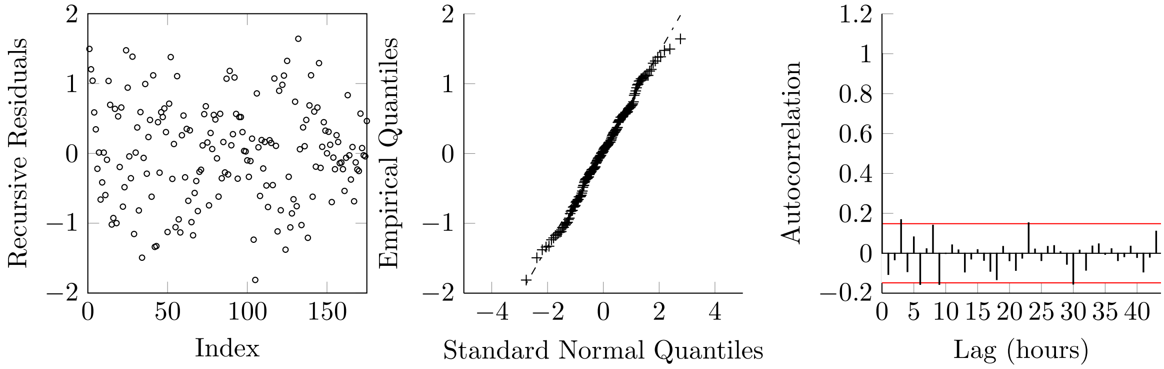

(b)

Fig. 23. Recursive residuals calculated for the time series shown in Figure 9 calculated at the posterior median estimates obtained under a) the LNA and b) the BDA. The left column shows the recursive residuals against index. The centre column gives a qq-plot of the residuals with no significant deviation from normality in both cases ($p < 0.05$ according to a Kolmogorov-Smirnov test for normality). The right column gives the autocorrelation of the residuals with the shaded region depicting the 95% envelopes of a white noise process.

References

Anderson, D F and Kurtz, T G. (2011). Continuous time Markov chain models for chemical reaction networks. In: *Design and Analysis of Biomolecular Circuits*. Springer, pp. 3–42.

Andrieu, C, Doucet, A and Holenstein, R. (2009). Particle Markov chain Monte Carlo for efficient numerical simulation. In: *Monte Carlo and quasi-Monte Carlo methods 2008*. Springer, pp. 45–60.

Andrieu, C, Doucet, A and Holenstein, R. (2010). Particle Markov chain Monte Carlo methods. *Journal of the Royal Statistical Society: Series B (Statistical Methodology)* **72**(3), 269–342.

Barbour, A D. (1974). On a functional central limit theorem for Markov population processes. *Advances in Applied Probability*, 21–39.

Boys, R J and Giles, P R. (2007). Bayesian inference for stochastic epidemic models with time-inhomogeneous removal rates. *Journal Of Mathematical Biology* **55**(2), 223–247.

Chesson, P. (1978). Predator-prey theory and variability. *Annual Review of Ecology and Systematics* **9**, 323–347.

Doucet, A., Godsill, S. and Andrieu, C. (2000). On sequential Monte Carlo sampling methods for Bayesian filtering. *Statistics and computing* **10**(3), 197–208.

Fearnhead, P, Giagos, V and Sherlock, C. (2014). Inference for reaction networks using the linear noise approximation. *Biometrics* **70**(2), 457–466.

Finkenstädt, B, Woodcock, D J, Komorowski, M, Harper, C V, Davis, J R E, White, M R H and Rand, D A. (2013). Quantifying intrinsic and extrinsic noise in gene transcription using the linear noise approximation: An application to single cell data. *The Annals of Applied Statistics* **7**(4), 1960–1982.

Gardiner, C W. (1985). *Handbook of stochastic methods for physics, chemistry and the natural sciences*. Springer Berlin.

Gillespie, D T. (2000). The chemical Langevin equation. *The Journal of Chemical Physics* **113**, 297.

Golightly, A and Wilkinson, D J. (2005). Bayesian inference for stochastic kinetic models using a diffusion approximation. *Biometrics* **61**(3), 781–788.

Golightly, A and Wilkinson, D J. (2011). Bayesian parameter inference for stochastic biochemical network models using particle Markov chain Monte Carlo. *Interface Focus* **1**(6), 807–820.

Green, P J. (1995). Reversible jump Markov chain Monte Carlo computation and Bayesian model determination. *Biometrika* **82**(4), 711–732.

Heron, E A, Finkenstädt, B and Rand, D A. (2007). Bayesian inference for dynamic transcriptional regulation; the Hes1 system as a case study. *Bioinformatics* **23**(19), 2596.

Jenkins, D J, Finkenstädt, B and Rand, D A. (2013). A temporal switch model for estimating transcriptional activity in gene expression. *Bioinformatics* **29**(9), 1158–1165.

Kalman, R E. (1960). A new approach to linear filtering and prediction problems. *Journal of Fluids Engineering* **82**(1), 35–45.

Kitagawa, G. (1996). Monte Carlo filter and smoother for non-Gaussian nonlinear state space models. *Journal of Computational and Graphical Statistics* **5**(1), 1–25.

Komorowski, M, Finkenstädt, B, Harper, C V and Rand, D A. (2009). Bayesian inference of biochemical kinetic parameters using the linear noise approximation. *BMC Bioinformatics* **10**(1), 343.

Kurtz, T G. (1970). Solutions of ordinary differential equations as limits of pure jump Markov processes. *Journal of Applied Probability* **7**(1), 49–58.

Kurtz, T G. (1971). Limit theorems for sequences of jump Markov processes approximating ordinary differential processes. *Journal of Applied Probability* **8**(2), 344–356.

McLachlan, G and Peel, D. (2004). *Finite mixture models*. John Wiley & Sons.

van Kampen, N G. (1961). A power series expansion of the master equation. *Canadian Journal of Physics* **39**(4), 551–567.

Wallace, E W J, Gillespie, D T, Sanft, K R and Petzold, L R. (2012). Linear noise approximation is valid over limited times for any chemical system that is sufficiently large. *Systems Biology, IET* **6**(4), 102–115.

Wilkinson, D J. (2011). *Stochastic modelling for systems biology*, Volume 44. CRC press.

[]
